# Survival dimensionality reduction (SDR): development and clinical application of an innovative approach to detect epistasis in presence of right-censored data

**DOI:** 10.1186/1471-2105-11-416

**Published:** 2010-08-06

**Authors:** Lorenzo Beretta, Alessandro Santaniello, Piet LCM van Riel, Marieke JH Coenen, Raffaella Scorza

**Affiliations:** 1 Referral Center for Systemic Autoimmune Diseases, Fondazione IRCCS Ca' Granda Ospedale Maggiore Policlinico and University of Milan, Milan, Italy; 2 Department of Rheumatology, Radboud University Nijmegen Medical Centre, Nijmegen, The Netherlands; 3 Department of Human Genetics, Radboud University Nijmegen Medical Centre, Nijmegen, The Netherlands; 4 On behalf of the Dutch Rheumatoid Arthritis Monitoring (DREAM) registry

## Abstract

**Background:**

Epistasis is recognized as a fundamental part of the genetic architecture of individuals. Several computational approaches have been developed to model gene-gene interactions in case-control studies, however, none of them is suitable for time-dependent analysis. Herein we introduce the Survival Dimensionality Reduction (SDR) algorithm, a non-parametric method specifically designed to detect epistasis in lifetime datasets.

**Results:**

The algorithm requires neither specification about the underlying survival distribution nor about the underlying interaction model and proved satisfactorily powerful to detect a set of causative genes in synthetic epistatic lifetime datasets with a limited number of samples and high degree of right-censorship (up to 70%). The SDR method was then applied to a series of 386 Dutch patients with active rheumatoid arthritis that were treated with anti-TNF biological agents. Among a set of 39 candidate genes, none of which showed a detectable marginal effect on anti-TNF responses, the SDR algorithm did find that the rs1801274 SNP in the FcγRIIa gene and the rs10954213 SNP in the IRF5 gene non-linearly interact to predict clinical remission after anti-TNF biologicals.

**Conclusions:**

Simulation studies and application in a real-world setting support the capability of the SDR algorithm to model epistatic interactions in candidate-genes studies in presence of right-censored data.

Availability: http://sourceforge.net/projects/sdrproject/

## Background

The complex nature of human disease has long been recognized and, with the exception of a limited number of examples which follow the rules of mendelian inheritance patterns, common disease results from the poorly understood interaction of genetic and environmental factors [1,2]. At the same time, gene-gene interactions that do not result in linearity between genotype and phenotype (*epistasis*), may involve several genes at time, dramatically increasing the complexity of the phenomenon. Epistasis can either be defined from a biological point of view as deviations from the simple inheritance patterns observed by Mendel [3] or, from a mathematical point of view, as deviations from additivity in a linear statistical model [4].

The study of statistical epistasis by traditional parametric models is challenging and hindered by several limitations. These include, the problem of the sparseness of data into the multidimensional space [5], the loss of power when adjusting for multiple testing to decrease type I error [6,7], the loss of power in presence of multicollinearity [8] or genetic heterogeneity [1]. To address these issues, several non-parameteric multi-locus methods, essentially based on machine-learning techniques, have been developed and/or applied to genetic association studies with positive results [9]. The application of data mining algorithms to detect non-linear high-order interactions in the context of survival analysis is more complex and thus far limited to a few examples [10-12]. However, the effective ability of these algorithms to model gene-gene interactions and their power to detect epistasis in survival analysis has yet to be determined.

At least two points in modelling non-linear interactions in survival analysis should be taken into account. The first, is the proper way to handle censored data, that is those cases for whom the outcome has not yet happened at the end of the observation time (*survival time*) or who did not have the event until the end of study (including lost cases and missing data), which are commonly referred to as *right-censored cases *[13]. The second, is the optimal performance measure to be used in assessing a learned model in survival analysis. In this paper we present an extension of the multifactor dimensionality reduction (MDR) algorithm [14,15], to detect and characterize epistatic interactions in the context of survival analysis which was specifically designed to address the abovementioned issues. Censored data were directly handled by estimating individual multilocus cells survival functions by the Kaplan-Meier method [16]. Multilocus genotypes were then pooled into high-risk and low-risk groups whose predictive accuracy was evaluated by the Brier score for censored samples proposed by Graf *et al *[17].

The power of the method we propose was at first evaluated in lifetime simulated datasets with epistatic effects which belonged to the most common survival distributions and with different degrees of right-censorship. The method was then applied to identification of single-nucleotide polymorphisms (SNPs) associated with responses to anti-tumor necrosis factor (TNF) agents in patients with rheumatoid arthritis (RA) and active disease.

The notion of pharmacogenetics is not anew in RA and several candidate-gene studies have demonstrated a genetically-based individual variability to treatment with methotrexate or anti-TNF therapy [18-20]. However, there is no consensus at present as to whether pharmacogenomics will allow prediction of anti-TNF therapy efficacy in RA. So far pharmacogenomics studies in RA have produced conflicting results and population stratification and linkage disequilibrium have been cited as potential causes for the inability to replicate results of genetic association studies [21]. Yet, as demonstrated by Greene *et al *[22] when main effects fail to replicate, gene-gene interaction analysis should also be considered as a potential source of variance.

## Methods

### Description of the survival dimensionality reduction (SDR) algorithm

The core of the SDR algorithm is the classification procedure used to label as "high-risk" or "low-risk" the multilocus cells that result from gene-gene interaction. This procedure will be used both for feature selection and for model validation as described in the forthcoming sections.

#### SDR assignments and evaluation

**Figure 1:**
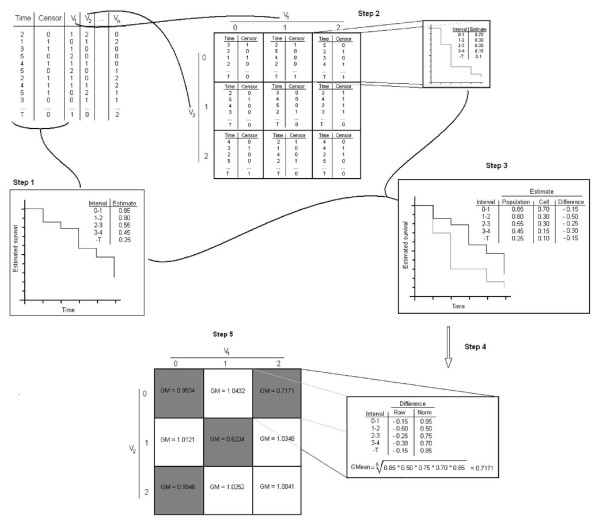
**Survival Dimensionality Reduction (SDR) process**. Step 1, survival estimates are calculated in the whole population by the Kaplan-Meier method. Step 2, survival estimates are computed in each multidimensional cell. Step 3, time-point differences in survival estimates between the whole population and each multidimensional cell are calculated. Step 4, time-point differences are normalized to 1 to take into account negative values and then averaged via the geometric mean (GM). Step 5, individual data from multidimensional cells with GM ≤ 1 ("high-risk") are pooled and compared via log rank-test statistic, to individual data from multidimensional cells with GM > 1 ("low-risk").

The SDR procedure for classification is illustrated in Figure 1 and it involves 5 steps.

*Step 1*: We firstly calculate by the Kaplan-Meier method [16] the survival estimates *Ŝ(t) *for the whole population in a dataset or dataset partition:

where, *n*_*i *_is the number of cases "at risk" of an event prior to *t*_*i*_, and *δ*_*i*_, is the number of events at time *t*_*i*_.

*Step 2: W*e then select *n *discrete variables from the dataset and represent all the possible multidimensional cells resulting from their interaction. For each multidimensional cell, survival estimates at each time interval *Ŝ*_*c*_*(t*_*i*_*) *are calculated as described above.

*Step 3: *The difference *D*_*c*_*(t*_*i*_*) *between the multilocus cell survival estimates and the whole population survival estimates, is calculated for each time interval *t*_*i*_:

*Step 4: *All the *D*_*c*_*(t*_*i*_*) *for each multilocus cells are then averaged. As the product-limit estimator is *de facto *a geometric progression, *D*_*c*_*(t*_*i*_*) *are averaged via the geometric mean (GM) rather than via the algebraic mean. As it is impossible to calculate the geometric with zero or negative data point values, these should be transformed to a meaningful equivalent positive number; being -1 <*D*_*c*_*(t*_*i*_*) *< 1, transformation is made adding 1 to any *D*_*c*_*(t*_*i*_*) *value. Considering a finite number *n *of time intervals and denoting *t*_*n *_as the survival time at the n^th ^time-interval we thus have:

*Step 5: C*ells with *GM*_*c*_*(t*_*n*_*) *≤ 1 are classified as "high-risk" and cells with *GM*_*c*_*(t*_*n*_*) *> 1 are classified as "low-risk". Examples from high-risk cells are pooled into one group and those from low-risk cells into another.

Once dimensionality has been reduced to one dimension, SDR predictions may be evaluated via different fitness measure. Herein, we employed the Brier score for censored data. The Brier score is a metric widely used for predicting the inaccuracy of a model and in its modified version proposed by Graf *et al *[17], it can incorporate censored samples. The Brier score BS(t) for censored samples for a given t > 0, is defined as:

where *Ĝ(t) *denotes the Kaplan-Meier estimate of the censoring distribution *G(t) *which is based on the observations (*t*_*i*_, 1 - *δ*_*i*_) and *I *stands for the indicator function. *BS(t) *depends on time *t*, hence it makes sense to use the integrated Brier score *(IBS) *as an overall measure for the prediction of the model at all times:

The lower the IBS the less inaccurate or, conversely, the more precise the prediction is.

#### Feature selection and model validation (k-fold Cross-Validation)

From a lifetime dataset, relevant features are extracted and validated via the k-fold cross-validation method, as described by Ritchie and co-workers [14].

For *feature selection*, the dataset is equally partitioned in *k *mutually-exclusive testing sets; one *k *set is retained for model validation whilst the remaining *k-1 *parts of the dataset are used as a training set. This process is then repeated *k*-times with each of the *k *testing samples never included in the feature selection process. In every training set, all the possible combinations of *n-*variables and the multilocus cells that result from their interactions are represented into the multidimensional space. SDR models are then iteratively built for each combination of *n-*variables and training IBS scores are calculated. For each *n-*combination of variables, the *k *training IBS are then averaged and the *n-*combination yielding the lowest mean IBS is selected and considered for model validation.

In the *model validation *phase, SDR assignments for the best n-combination of variables are determined in every training set. On the basis of training assignments, the instances from the corresponding k testing sets are labelled as "high-risk" or "low-risk". From these labels, a cross-validated IBS is then calculated; for this purpose, instead of mathematically averaging the *k *testing IBS, we merged the testing instances in a meta-analysis-based on individual patient data-fashion [23]. Let T_1_, T_2, --- _T_k _be the *k *testing sets, the individual patients data together by their assigned labels are merged sort to produce a larger T_M _testing set. The IBS (henceforth labelled as *meta-IBS*) is computed in the T_M _set and the *n-*combination yielding the lowest *meta-IBS *is then chosen as the final model. This way both feature selection and model validation are used to determine the best epistatic model in lifetime datasets. Working on the merged dataset (T_M_), we: 1) still ensure the independence of the testing sets, as testing assignments are calculated during the *feature selection *phase; 2) reduce the bias in the calculation of the BS(t), as the *Ĝ(t) *and *G(t) *weights used in the BS(t) formula would otherwise be unreliably estimated in testing sets of limited size; 3) avoid to solely rely on a measure of central tendency (e.g. the mean) to estimate the predictive accuracy of our model, utterly ignoring any measure of variance.

### Data simulation and power calculation

Simulated epistatic datasets were modelled upon five different survival distributions, described by the logistic-exponential equation [24]; exponential (EXP), bathtub-shaped failure rate (BT), upside-down bathtub-shaped failure rate (UBT), decreasing failure rate (DFR) and increasing failure rate (IFR):

where, *S(t) *is the logistic-exponential survival distribution, *t *is the survival time, *λ *is a positive scale parameter and *κ *is a positive shape parameter and *θ *is a ≥ 0 parameter that shifts the distribution to the left. λ, *κ *and *θ *were adjusted so that the cumulative prevalence of the event at the end of the observation time *t*_*n *_*or K(t*_*n*_*)*, was equal to an arbitrary value of 0.750 (see Additional file 1, Table S1). For data simulation *t*_*n *_was set to 5 time units.

According to Culverhouse *et al *[25], we then generated different epistatic models for two biallelic SNPs A and B, both in Hardy-Weinberg equilibrium (HWE) and with minor allele frequency (MAF) = 0.2, so that *K(t*_*n*_*) *= *K*_*A *_= *K*_*B*_, where *K*_*A *_and *K*_*B *_are the marginal penetrances for SNP A and SNP B. As these models were adjusted to fit the cumulative prevalence at *t*_*n*_, their broad-sense heritability (H^2^) was considered to be a *cumulative estimate *of H^2 ^at *t*_*n *_or *H*^*2*^*(t*_*n*_*)*. For data simulation *H*^*2*^*(t*_*n*_*) *was set to 0.10, 0.15, 0.20 or 0.25.

Time-point multilocus genotype penetrances- henceforth labelled as *(Genotype)t*_*I *_-, were adjusted so that the cumulative prevalence at each time-point *K(t*_*i*_*) *would fit the survival distribution. We assumed that the relation between SNP A and SNP B is purely epistatic at each time-interval *t*_*i*_, that is *K(t*_*i*_*) *= *K*_*A*_*(t*_*i*_*) *= *K*_*B*_*(t*_*i*_*) *with 0 <*t*_*i *_≤ *t*_*n*_, and that the two-locus model is always proportional at the different *t*_*i*_. Hence, applying the product-limit estimation, we obtain:

where *Kt*_*i *_is the time-point prevalence at *t*_*i *_*and; t*_*i *_≤ *t*_*n*_.

From *Kt*_*i *_we can calculate *(Genotype)t*_*i *_from the cumulative multilocus genotype penetrances [*Genotype(t*_*n*_*)*] previously used to compute *K(t*_*n*_*) *and *H*^*2*^*(t*_*n*_*):*

Similarly, time-point cumulative estimated multilocus genotype penetrances [*Genotype(t*_*i*_*)*] are proportional to *K(t*_*i*_*)*. These penetrances can be used to derive the time-point cumulative estimated *H*^*2 *^or *H*^*2*^*(t*_*i*_*)*.

Once *(Genotype)t*_*i *_values had been calculated, a population of 65000 individuals was built considering all the time-intervals *0 < t*_*i *_≤ *t*_*n*_. Herein, from 5 survival distributions with 3 different *H*^*2*^*(t*_*n*_*) *and two *t*_*n *_we obtained 40 populations where the outcome was related to the epistatic interaction between SNP A and SNP B (see Additional file 1). To each of these populations 13 unrelated SNPs in HWE, with MAF ranging from 0.1 to 0.5 were added. An additional 5% censoring/year was also added to account for hypothetical non-event related causes of withdrawal from observation.

From the simulated populations we finally randomly draw 100 samples of 200 cases and 200 controls (e.g. 50% censorship) or 120 cases and 280 controls (e.g. 70% censorship). A total of 4,000 datasets were then generated for simulation. Power was estimated as the number of times SDR correctly identified the two functional SNPs out of 100 datasets/model/degree-of-censorship. Datasets can be obtained upon request from the authors.

### Application of the SDR algorithm to the RA dataset

The SDR algorithm was then tested in a real-world dataset which consists of previously unpublished data about 386 Dutch patients with (1) a diagnosis of RA according to ACR criteria [26], (2) a disease activity score (DAS28) >3.2 [27] and (3) previous treatment with at least two other anti-rheumatics including methotrexate (MTX) at an optimal dose (maximum dose of 25 mg/week) or intolerance for MTX, that underwent treatment with anti-TNFα agents. These patients were extracted from the Dutch Rheumatoid Arthritis Monitoring (DREAM) registry [28] and genotyped for 39 candidate SNPs and evaluated every 3 months to ascertain whether they had reached a clinical remission, defined as a DAS28 ≤ 2.6 [27]. Genotyping details can be found in: Pavy *et al *[29], Coenen *et al *[30], Toonen *et al *[31] and Alizadeh *et al *[32]. The choice of the studied SNPs was motivated by results from previous association and/or pharmacogenomics studies in RA [18-20,33]. A detailed list of the analysed genetic variants is provided in Additional file 2, Table S1. The k-nearest-neighbour method was used to impute genotypes with missing data <10% [34]. The open-source Orange data mining software (available at: http://www.ailab.si/orange) was used for imputation. Overall the right-censorship of this dataset was 68%; 5-fold cross-validation was used for the SDR analysis. An empirical *P*-value for the SDR results was calculated by performing 100-fold permutation testing [35]. A whole SDR analysis, up to the 3^rd ^dimension was conducted in the permutated datasets during the permutation procedure.

For all the analyses a modified version of the freely available SDR algorithm written in Python http://sourceforge.net/projects/sdrproject/ was used.

## Results

### Simulated datasets

**Figure 2:**
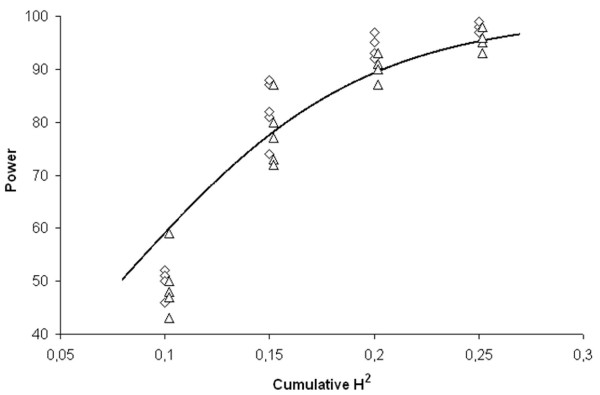
**Trend of the power for the survival dimensionality reduction algorithm in the different models**. Relationship between power and cumulative broad-sense heritability (H^2^) according to the different degrees of censorship of the sample datasets (squares = 70%; triangles = 50%). The line represents the power estimated by the logistic function (R^2 ^= 0.846).

The penetrance functions for the simulated datasets are reported in Additional file 1; as it can be observed, time-point H^2 ^across the different models were consistently low, with a median of 0.017 (interquartile range [IQR]: 0,011 - 0.025). Power for the SDR algorithm to correctly identify the causative pair of SNPs in the 20 simulated survival models with 2 different degrees of censorship is reported in Table 1. Overall, the median power across all the datasets was 87.5% (IQR, 62.25% - 95%). The relationship between *H*^*2*^*(t*_*n*_*) *and power followed a direct logistic distribution as shown in Figure 2, (overall, R^2 ^= 0.846; 50% censoring, R^2 ^= 0.886; 70% censoring, R^2 ^= 0.870). Moreover, the power resulted to be independent from the survival distribution the sample was withdrawn from whilst it was inversely related to the degree of censorship. The *H*^*2*^*(t*_*n*_*) *values we employed correspond to mild-to-moderate hazard ratios (HR) for high-risk vs low-risk combinations in the different epistatic models: HR = 1.38 for *H*^*2*^*(t*_*n*_*) *= 0.10, HR = 1.5 for *H*^*2*^*(t*_*n*_*) *= 0.15, HR = 1.58 for *H*^*2*^*(t*_*n*_*) *= 0.20 and HR = 1.69 for *H*^*2*^*(t*_*n*_*) *= 0.25. Altogether these results suggest that SDR has a satisfactory power to identify a pair of causative genes in purely epistatic lifetime models with mild-to-moderate effect size and for which the proportionality of hazards holds.

**Table 1:** Power for the Survival Dimensionality Reduction (SDR) algorithm in models with cumulative prevalence K(t_n_) = 0.750

		**H**^**2**^**(t**_**5**_**)**			
		
Model	RCR	0.10	0.15	0.20	0.25
**UBT**	50%	52	81	95	97
	70%	59	73	93	93
**DFR**	50%	52	87	97	98
	70%	48	77	87	96
**IFR**	50%	51	88	92	99
	70%	50	87	91	98
**BT**	50%	46	74	93	97
	70%	43	72	91	95
**EXP**	50%	50	82	95	99
	70%	47	80	90	96

Power of the SDR algorithm in simulated datasets modelled using the 5 classes of survival distribution described by the logistic-exponential equation. Different degrees of right-censoring rate (RCR), and cumulative heritability at the survival time [H^2^(t_5_)] are considered. UBT, upside-down bathtub-shaped failure rate; DFR, decreasing failure rate; IFR, increasing failure rate; BT bathtub-shaped failure rate; EXP, exponential.

### RA dataset

The RA datasets comprises 386 anti-TNFα-treated patients, aging 45 ± 13.7 (mean ± standard deviation) at the onset of disease; 78.3% of patients tested positive for the rheumatoid factor and 262 (67.9%) were females. One-hundred-forty-five patients (37.6%) were treated with adalimumab, 201 (52.1%) with infliximab and 40 (10.4%) with etanercept; overall 346 patients (89.6%) were treated with anti-TNF antibodies (e.g. adalimumab and infliximab). DAS28 at the beginning of therapy was 5.64 ± 1.09. Clinical remission, based on DAS28, was observed in 123 cases (31.8%).

Details about the genotyped SNPs along with their frequencies in the studied population are reported in Additional file 2, Table S1; all the SNPs were in HWE. None of the single SNPs showed a statistically significant association with response to anti-TNF agents, either under a dominant or recessive model, as illustrated in Additional file 2, Table S2 (log-rank-associated p values with 1 degree of freedom, corrected for the number of comparison by Bonferroni adjustment >0.05).

**Figure 3:**
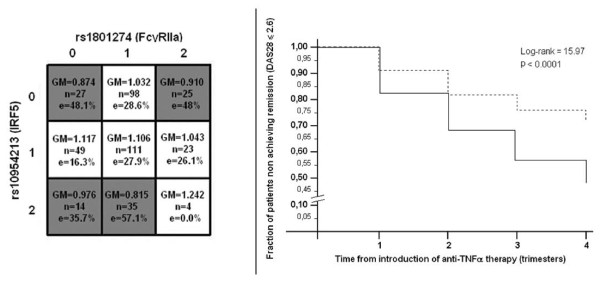
**Survival dimensionality reduction (SDR) model for the rheumatoid arthritis dataset**. a. Multidimensional matrix for the single nucleotide polymorphisms (SNPs) interaction; GM, geometric mean of the differences; n, number of cases; e, percentage of events. b. Kaplan-Meier estimated survival patterns associated with "high-" and "low-risk" assignments. P values are those associated with a chi-square distribution with 1 degree of freedom.

The SDR algorithm sorted out two-way interaction model, involving the rs1801274 (Fc gamma receptor 2a, FcγRIIa) and the rs10954213 (interferon regulatory factor 5, IRF5) SNPs, as the most predictive for responses to anti-TNF therapy in patients with active RA. Table 2 shows the full analysis conducted by the SDR algorithm in the RA datasets. As expected, the model overfits in the training population as the number of SNPs included in the model increases. Yet, cross-validation prevented over-fitting as the minimum *meta-IBS *was observed for the 2-way interaction, that was thus chosen as the best epistatic model. This model was significant at the 0.05 threshold after 100-fold permutation testing. Figure 3a summarizes the multilocus cells for the rs1801274 × rs10954213 interaction along with SDR "high-" and "low-risk" assignments (e.g. "responders" and "not responders" to therapy). This interaction had the typical non-linear behaviour of epistatic model. Plotting this SDR assignments we can observe that patients labelled as "responders" achieved earlier and higher rates of clinical remission after anti-TNF therapy compared to patients labelled as "non-responders" (figure 3b) [36].

**Table 2:** Survival dimensionality reduction (SDR) model for the rheumatoid arthritis (RA) dataset.

		IBS		
			
n-way	SNPs (genes) in each dimension	Training	Testing	p
1	rs2327832 (TNFAIP3, OLIG3)	0.263	0.2366	-
**2**	**rs1801274 (FcγRIIa), rs10954213 (IRF5)**	**0.2339**	**0.2354**	**<0.05**
3	rs1801274 (FcγRIIa), rs10954213 (IRF5), rs3761847 (TRAF1)	0.2219	0.2393	-

Selection of the best combination of attributes by the SDR method. The model with the minimum testing IBS value in the cross-validated testing sets is indicated in boldface type. p values associated with the log-rank test statistic calculated by the 100-fold permutation test. SNP, single nucleotide polymorphism.

## Discussion

In the present paper we introduce SDR, an algorithm specifically conceived to detect non-linear gene-gene interactions in presence of right-censored data. The need for such a bioinformatics tool comes from the observation that several studies in the medical field deal with the loss of data during the period of study, and that methods that do not take into account censored data give upwardly biased estimates of failure and/or suffer from information loss due to reduced sample size. Cox regression [37], the most popular statistical technique used to analyse time-to-event multivariate model, is not adequate to detect non-linearities either. Indeed, to properly model interaction in Cox regression, the user should have *a priori *knowledge of the variable relationships and may need to enter nonlinear transforms of the predictors, but this is often a trial and error approach. Also, the number of polynomial terms needed to model complex interactions is inflated as the number of predictors grows, increasing standard errors and, thus, type I error [6,7].

Using simulated epistatic lifetime datasets, we demonstrated that the SDR algorithm retains a fully satisfactory power to sort out a set of causative genes with mild-to-moderate epistatic effect size from a pool of candidate genes. These results have been accomplished by the intrinsic properties of the SDR methodology. Firstly, SDR is non-parametric, in the sense that it is not necessary to make *a priori *assumptions about the underlying interaction model. Also, SDR requires no assumptions concerning the nature or shape of the underlying survival distribution. Secondly, SDR performs well even in datasets where the right-censorship rate is high (up to 70% of cases in our simulation). Notably, when we run MDR ignoring censorship on the same simulated datasets we observed a dramatic reduction in the power to detect the causative pair of genes, that ranged from 5% to more than 90% (results not shown). Beside power, an additional advantage of SDR is that, combining cross-validation with permutation testing, the chance of false-positive findings is minimized [14]. Yet, to generate the simulated datasets we required the hazards among multilocus cells to be proportional along the lifetime distribution. Hence, further simulations are needed to ascertain whether SDR is suitable for situations where this assumption does not hold, as for instance in case of additive hazard models. Similarly, further simulations are needed to establish SDR performance in larger datasets, in presence of genetic heterogeneity, linkage disequilibrium, or different ranges of MAF.

SDR is, *de facto*, an extension of the MDR algorithm optimized to analyse lifetime distributions, hence it suffers from similar limitations [14] and it shares some peculiarities with the latter. Namely, the power of SDR is influenced by the epistatic effect size, which is strictly related to the (cumulative) heritability of the model [38]. Moreover, as with MDR, the biological significance of SDR models may be difficult to interpret due to the non-linear distribution of high-risk and low-risk cells across the multidimensional space [39]. Finally, SDR cannot make predictions when multilocus cells contain no data and GM estimates may be upwardly or downwardly inflated when multilocus cells contain few data.

Having demonstrated the capability of SDR to detect gene-gene interactions in lifetime datasets, we applied the algorithm to a population of patients with active RA to identify epistatic interactions that may affect time-related responses to anti-TNF biological agents. We did show that among a set of 39 candidate-gene loci, none of which had a detectable marginal effect on the outcome variable, the non-linear interaction between the rs1801274 (FcγRIIa) and the rs10954213 (IRF5) SNPs significantly predicted the responses to anti-TNF therapy.

Whilst it is difficult to dissect the biological meaning of statistical epistatic models [39], it should be noted that several lines of evidence support a role for Fcγ receptors and IRF5 in rheumatoid arthritis and TNF driven processes. Associations between IRF5 polymorphisms and RA have been described in different populations [40] and a recent genome-wide association study (GWAS) showed that the rs4728142 variant in the IRF5 gene, in tight linkage disequilibrium with rs10954213, is strongly associated with RA susceptibility [41]. Of interest, functional experiments demonstrated that the rs10954213 SNP significantly alters IRF5 mRNA expression [42]. The association between IRF5 gene and RA may be linked, at least in part, to the ability of IRF5 to regulate the secretion of pro-inflammatory cytokines. Indeed, Takaoka *et al *[43] using mouse models deficient in the IRF5 gene, showed that IRF5 is generally involved downstream of the toll-like receptor (TLR)-MyD88 signalling pathway for gene induction of TNFα and other cytokines relevant to the pathogenesis of RA. Of interest, the use of anti-TNF agents was shown to decrease TLRs expression on different cellular types [44,45]. Similarly to IRF5, the FcγRIIa is involved in TNFα production in the rheumatoid synovia, as observed by Clavel and co-workers [46]. The interaction between the genetic variants of IRF5 and Fcγ receptors could thus influence TNFα production and/or availability, affecting the clinical response to anti-TNF agents. Additionally, as postulated by Cañete *at al *[47], polymorphisms of the FcγRIIa may alter the clearance rate of anti-TNF antibodies modulating plasma concentrations and consequently their biological effect in subjects with active RA. Theoretically, this effect should not restricted only to anti-TNF antibodies, as also anti-TNFα receptors (e.g. etanercept) contain a Fc portion of IgG_1 _capable of binding to FCγ receptors to produce biological effects, such as antibody-dependent cell-mediated cytotoxicity [48].

## Conclusions

Herein we introduced SDR, an innovative algorithm to detect epistasis in lifetime datasets. Simulation studies and application in a real-world setting, demonstrate the capability of SDR to detect non linear gene-gene interactions in studies aimed at evaluating the effect of candidate genes on time-dependent outcomes. Further studies are necessary to evaluate its applicability in large-scale datasets as well.

## Supplementary Material

Additional file 1 **Epistatic models and simulation specifics**. The file contains the settings used to generate the five survival distributions upon which epistatic models were modelled. For each epistatic model, ttime-point and cumulative multilocus genotype penetrances are reported, along with the time-point and the cumulative broad-sense heritability and prevalence of the event. Click here for file

Additional file 2 **Genotype frequencies and univariate analysis in the rheumatoid arthritis (RA) dataset**. The file lists in tabular form the single nucleotide polymorphisms (SNPs) included in the RA case-control study. It also reports the associations by the log-rank test statistics under the dominant and recessive model between the studied SNPs and the occurrence of clinical remission after therapy with anti-TNF agents. Click here for file
