# Supplementary material for: Survival dimensionality reduction (SDR): development and clinical application of an innovative approach to detect epistasis in presence of right-censored data
Source: BMC Bioinformatics. 2010 Aug 6;11:416. doi: 10.1186/1471-2105-11-416 (PMC2928804; doi:10.1186/1471-2105-11-416)
Supplement: Additional file 1 — Epistatic models and simulation specifics. The file contains the settings used to generate the five survival distributions upon which epistatic models were modelled. For each epistatic model, ttime-point and cumulative multilocus genotype penetrances are reported, along with the time-point and the cumulative broad-sense heritability and prevalence of the event. [file 1471-2105-11-416-S1.DOC]

**Table S1 – Settings employed to generate the different populations used for simulation**

| **Model number** | **K** | **λ** | **θ** |
| --- | --- | --- | --- |
| 1-8 | 3 | 0.149 | 1 |
| 9-16 | 1.5 | 0.182 | 20 |
| 17-24 | 0.5 | 0.6081 | 5 |
| 25-32 | 0.5 | 0.64 | 0.9 |
| 33-40 | 1 | 0.277 | - |

**Model 1 & 2– Upside-down bathtub-shaped survival distribution; H^2^(5) = 0.10; K(5) = 0.750**

|  | **Time-Point** | | | | |
| --- | --- | --- | --- | --- | --- |
|  | **5** | **4** | **3** | **2** | **1** |
| **K** |  |  |  |  |  |
| Time-point | 0,2419 | 0,2420 | 0,2419 | 0,2420 | 0,2419 |
| Cumulative | 0,7497 | 0,6698 | 0,5644 | 0,4254 | 0,2419 |
| **H2** |  |  |  |  |  |
| Time-point | 0,0109 | 0,0109 | 0,0109 | 0,0109 | 0,0109 |
| Cumulative | 0,1003 | 0,0678 | 0,0435 | 0,0249 | 0,0109 |
| **Penetrance/genotype** |  |  |  |  |  |
| **Time-point** |  |  |  |  |  |
| AABB | 0,2181 | 0,2181 | 0,2181 | 0,2181 | 0,2181 |
| AABb | 0,2826 | 0,2826 | 0,2826 | 0,2826 | 0,2826 |
| AAbb | 0,2984 | 0,2985 | 0,2984 | 0,2984 | 0,2984 |
| AaBB | 0,2894 | 0,2894 | 0,2894 | 0,2894 | 0,2893 |
| AaBb | 0,1623 | 0,1623 | 0,1623 | 0,1623 | 0,1622 |
| Aabb | 0,1210 | 0,1210 | 0,1210 | 0,1210 | 0,1210 |
| aaBB | 0,2445 | 0,2446 | 0,2445 | 0,2446 | 0,2445 |
| aaBb | 0,2287 | 0,2288 | 0,2287 | 0,2288 | 0,2287 |
| aabb | 0,3061 | 0,3062 | 0,3061 | 0,3062 | 0,3061 |
| **Penetrance/genotype** |  |  |  |  |  |
| **Cumulative** |  |  |  |  |  |
| AABB | 0,6760 | 0,6040 | 0,5089 | 0,3836 | 0,2182 |
| AABb | 0,8760 | 0,7826 | 0,6595 | 0,4970 | 0,2827 |
| AAbb | 0,9250 | 0,8264 | 0,6964 | 0,5248 | 0,2985 |
| AaBB | 0,8970 | 0,8014 | 0,6753 | 0,5089 | 0,2895 |
| AaBb | 0,5030 | 0,4494 | 0,3787 | 0,2854 | 0,1623 |
| Aabb | 0,3750 | 0,3350 | 0,2823 | 0,2128 | 0,1210 |
| aaBB | 0,7580 | 0,6772 | 0,5707 | 0,4301 | 0,2446 |
| aaBb | 0,7090 | 0,6334 | 0,5338 | 0,4023 | 0,2288 |
| aabb | 0,9490 | 0,8479 | 0,7144 | 0,5384 | 0,3063 |

**Model 3 & 4– Upside-down bathtub-shaped survival distribution; H^2^(5) = 0.15; K(5) = 0.750**

|  |  | **Time-point** | | |  |
| --- | --- | --- | --- | --- | --- |
|  | **5** | **4** | **3** | **2** | **1** |
| **K** |  |  |  |  |  |
| Time-point | 0,4138 | 0,3455 | 0,2349 | 0,1164 | 0,0362 |
| Cumulative | 0,7500 | 0,5735 | 0,3484 | 0,1483 | 0,0362 |
| **H^2^** |  |  |  |  |  |
| Time-point | 0,0363 | 0,0270 | 0,0156 | 0,0068 | 0,0029 |
| Cumulative | 0,1536 | 0,0687 | 0,0273 | 0,0087 | 0,0029 |
| **Penetrance/genotype** |  |  |  |  |  |
| **Time-point** |  |  |  |  |  |
| AABB | 0,3630 | 0,3031 | 0,2061 | 0,1021 | 0,0317 |
| AABb | 0,5032 | 0,4201 | 0,2856 | 0,1415 | 0,0440 |
| AAbb | 0,5103 | 0,4261 | 0,2897 | 0,1435 | 0,0446 |
| AaBB | 0,5148 | 0,4298 | 0,2922 | 0,1448 | 0,0450 |
| AaBb | 0,2378 | 0,1985 | 0,1350 | 0,0669 | 0,0208 |
| Aabb | 0,2069 | 0,1727 | 0,1174 | 0,0582 | 0,0181 |
| aaBB | 0,4182 | 0,3492 | 0,2374 | 0,1176 | 0,0366 |
| aaBb | 0,3912 | 0,3266 | 0,2221 | 0,1100 | 0,0342 |
| aabb | 0,5236 | 0,4372 | 0,2972 | 0,1472 | 0,0458 |
| **Penetrance/genotype** |  |  |  |  |  |
| **Cumulative** |  |  |  |  |  |
| AABB | 0,6580 | 0,5032 | 0,3057 | 0,1301 | 0,0317 |
| AABb | 0,9120 | 0,6974 | 0,4236 | 0,1804 | 0,0440 |
| AAbb | 0,9250 | 0,7074 | 0,4297 | 0,1829 | 0,0446 |
| AaBB | 0,9330 | 0,7135 | 0,4334 | 0,1845 | 0,0450 |
| AaBb | 0,4310 | 0,3296 | 0,2002 | 0,0852 | 0,0208 |
| Aabb | 0,3750 | 0,2868 | 0,1742 | 0,0742 | 0,0181 |
| aaBB | 0,7580 | 0,5796 | 0,3521 | 0,1499 | 0,0366 |
| aaBb | 0,7090 | 0,5422 | 0,3294 | 0,1402 | 0,0342 |
| aabb | 0,9490 | 0,7257 | 0,4408 | 0,1877 | 0,0458 |

**Model 5 & 6 – Upside-down bathtub-shaped survival distribution; H^2^(5) = 0.20; K(5) = 0.750**

|  |  | **Time-point** | | |  |
| --- | --- | --- | --- | --- | --- |
|  | **5** | **4** | **3** | **2** | **1** |
| **K** |  |  |  |  |  |
| Time-point | 0,4138 | 0,3455 | 0,2349 | 0,1164 | 0,0362 |
| Cumulative | 0,7500 | 0,5735 | 0,3484 | 0,1483 | 0,0362 |
| **H^2^** |  |  |  |  |  |
| Time-point | 0,0482 | 0,0363 | 0,0211 | 0,0088 | 0,0029 |
| Cumulative | 0,2053 | 0,0920 | 0,0366 | 0,0119 | 0,0029 |
| **Penetrance/genotype** |  |  |  |  |  |
| **Time-point** |  |  |  |  |  |
| AABB | 0,3559 | 0,2971 | 0,2020 | 0,1001 | 0,0311 |
| AABb | 0,5297 | 0,4422 | 0,3007 | 0,1489 | 0,0463 |
| AAbb | 0,4138 | 0,3455 | 0,2349 | 0,1164 | 0,0362 |
| AaBB | 0,5241 | 0,4376 | 0,2975 | 0,1474 | 0,0458 |
| AaBb | 0,1953 | 0,1631 | 0,1109 | 0,0549 | 0,0171 |
| Aabb | 0,3967 | 0,3312 | 0,2252 | 0,1116 | 0,0347 |
| aaBB | 0,4579 | 0,3823 | 0,2600 | 0,1288 | 0,0400 |
| aaBb | 0,3084 | 0,2575 | 0,1751 | 0,0867 | 0,0270 |
| aabb | 0,5501 | 0,4593 | 0,3123 | 0,1547 | 0,0481 |
| **Penetrance/genotype** |  |  |  |  |  |
| **Cumulative** |  |  |  |  |  |
| AABB | 0,6450 | 0,4932 | 0,2996 | 0,1276 | 0,0311 |
| AABb | 0,9600 | 0,7341 | 0,4459 | 0,1899 | 0,0463 |
| AAbb | 0,7500 | 0,5735 | 0,3484 | 0,1483 | 0,0362 |
| AaBB | 0,9500 | 0,7265 | 0,4413 | 0,1879 | 0,0458 |
| AaBb | 0,3540 | 0,2707 | 0,1644 | 0,0700 | 0,0171 |
| Aabb | 0,7190 | 0,5498 | 0,3340 | 0,1422 | 0,0347 |
| aaBB | 0,8300 | 0,6347 | 0,3856 | 0,1642 | 0,0400 |
| aaBb | 0,5590 | 0,4275 | 0,2597 | 0,1106 | 0,0270 |
| aabb | 0,9970 | 0,7624 | 0,4631 | 0,1972 | 0,0481 |

**Model 7 & 8 – Upside-down bathtub-shaped survival distribution; H^2^(5) = 0.25; K(5) = 0.750**

|  |  | **Time-point** | | |  |
| --- | --- | --- | --- | --- | --- |
|  | **5** | **4** | **3** | **2** | **1** |
| **K** |  |  |  |  |  |
| Time-point | 0,4139 | 0,3455 | 0,2349 | 0,1164 | 0,0362 |
| Cumulative | 0,7500 | 0,5735 | 0,3484 | 0,1483 | 0,0362 |
| **H^2^** |  |  |  |  |  |
| Time-point | 0,0589 | 0,0438 | 0,0256 | 0,0107 | 0,0029 |
| Cumulative | 0,2496 | 0,1121 | 0,0445 | 0,0143 | 0,0029 |
| **Penetrance/genotype** |  |  |  |  |  |
| **Time-point** |  |  |  |  |  |
| AABB | 0,3503 | 0,2924 | 0,1988 | 0,0985 | 0,0306 |
| AABb | 0,5412 | 0,4518 | 0,3072 | 0,1522 | 0,0473 |
| AAbb | 0,4138 | 0,3454 | 0,2348 | 0,1163 | 0,0362 |
| AaBB | 0,5357 | 0,4472 | 0,3040 | 0,1506 | 0,0468 |
| AaBb | 0,1716 | 0,1432 | 0,0974 | 0,0482 | 0,0150 |
| Aabb | 0,3967 | 0,3311 | 0,2251 | 0,1115 | 0,0347 |
| aaBB | 0,4579 | 0,3823 | 0,2599 | 0,1287 | 0,0400 |
| aaBb | 0,3084 | 0,2574 | 0,1750 | 0,0867 | 0,0270 |
| aabb | 0,5501 | 0,4592 | 0,3122 | 0,1546 | 0,0481 |
| **Penetrance/genotype** |  |  |  |  |  |
| **Cumulative** |  |  |  |  |  |
| AABB | 0,6350 | 0,4856 | 0,2950 | 0,1256 | 0,0306 |
| AABb | 0,9810 | 0,7502 | 0,4557 | 0,1940 | 0,0473 |
| AAbb | 0,7500 | 0,5735 | 0,3484 | 0,1483 | 0,0362 |
| AaBB | 0,9710 | 0,7425 | 0,4511 | 0,1920 | 0,0468 |
| AaBb | 0,3110 | 0,2378 | 0,1445 | 0,0615 | 0,0150 |
| Aabb | 0,7190 | 0,5498 | 0,3340 | 0,1422 | 0,0347 |
| aaBB | 0,8300 | 0,6347 | 0,3856 | 0,1642 | 0,0400 |
| aaBb | 0,5590 | 0,4275 | 0,2597 | 0,1106 | 0,0270 |
| aabb | 0,9970 | 0,7624 | 0,4631 | 0,1972 | 0,0481 |

**Model 9 & 10 – Decreasing failure rate survival distribution; H^2^(5) = 0.10; K(5) = 0.750**

|  | **Time-Point** | | | | |
| --- | --- | --- | --- | --- | --- |
|  | **5** | **4** | **3** | **2** | **1** |
| **K** |  |  |  |  |  |
| Time-point | 0,2411 | 0,2415 | 0,2419 | 0,2425 | 0,2432 |
| Cumulative | 0,7498 | 0,6704 | 0,5654 | 0,4267 | 0,2432 |
| **H2** |  |  |  |  |  |
| Time-point | 0,0104 | 0,0109 | 0,0109 | 0,0109 | 0,0109 |
| Cumulative | 0,1003 | 0,0684 | 0,0436 | 0,0249 | 0,0109 |
| **Penetrance/genotype** |  |  |  |  |  |
| **Time-point** |  |  |  |  |  |
| AABB | 0,2173 | 0,2176 | 0,2180 | 0,2186 | 0,2192 |
| AABb | 0,2816 | 0,2820 | 0,2826 | 0,2833 | 0,2841 |
| AAbb | 0,2973 | 0,2978 | 0,2984 | 0,2991 | 0,3000 |
| AaBB | 0,2883 | 0,2888 | 0,2893 | 0,2901 | 0,2909 |
| AaBb | 0,1617 | 0,1619 | 0,1622 | 0,1627 | 0,1631 |
| Aabb | 0,1205 | 0,1207 | 0,1210 | 0,1213 | 0,1216 |
| aaBB | 0,2436 | 0,2440 | 0,2445 | 0,2451 | 0,2458 |
| aaBb | 0,2279 | 0,2283 | 0,2287 | 0,2293 | 0,2299 |
| aabb | 0,3050 | 0,3055 | 0,3061 | 0,3069 | 0,3078 |
| **Penetrance/genotype** |  |  |  |  |  |
| **Cumulative** |  |  |  |  |  |
| AABB | 0,6760 | 0,6044 | 0,5098 | 0,3847 | 0,2192 |
| AABb | 0,8760 | 0,7832 | 0,6606 | 0,4985 | 0,2841 |
| AAbb | 0,9250 | 0,8270 | 0,6975 | 0,5264 | 0,3000 |
| AaBB | 0,8970 | 0,8019 | 0,6764 | 0,5105 | 0,2909 |
| AaBb | 0,5030 | 0,4497 | 0,3793 | 0,2863 | 0,1631 |
| Aabb | 0,3750 | 0,3353 | 0,2828 | 0,2134 | 0,1216 |
| aaBB | 0,7580 | 0,6777 | 0,5716 | 0,4314 | 0,2458 |
| aaBb | 0,7090 | 0,6339 | 0,5346 | 0,4035 | 0,2299 |
| aabb | 0,9490 | 0,8484 | 0,7156 | 0,5401 | 0,3078 |

**Model 11 & 12 – Decreasing failure rate survival distribution; H^2^(5) = 0.15; K(5) = 0.750**

|  |  | **Time-point** | | |  |
| --- | --- | --- | --- | --- | --- |
|  | **5** | **4** | **3** | **2** | **1** |
| **K** |  |  |  |  |  |
| Time-point | 0,2411 | 0,2415 | 0,2419 | 0,2425 | 0,2432 |
| Cumulative | 0,7498 | 0,6704 | 0,5654 | 0,4267 | 0,2432 |
| **H^2^** |  |  |  |  |  |
| Time-point | 0,0164 | 0,0164 | 0,0164 | 0,0163 | 0,0163 |
| Cumulative | 0,1536 | 0,1042 | 0,0668 | 0,0380 | 0,0163 |
| **Penetrance/genotype** |  |  |  |  |  |
| **Time-point** |  |  |  |  |  |
| AABB | 0,2115 | 0,2118 | 0,2122 | 0,2128 | 0,2134 |
| AABb | 0,2931 | 0,2936 | 0,2942 | 0,2949 | 0,2958 |
| AAbb | 0,2973 | 0,2978 | 0,2984 | 0,2991 | 0,3000 |
| AaBB | 0,2999 | 0,3004 | 0,3009 | 0,3017 | 0,3026 |
| AaBb | 0,1385 | 0,1388 | 0,1390 | 0,1394 | 0,1398 |
| Aabb | 0,1205 | 0,1207 | 0,1210 | 0,1213 | 0,1216 |
| aaBB | 0,2436 | 0,2440 | 0,2445 | 0,2451 | 0,2458 |
| aaBb | 0,2279 | 0,2283 | 0,2287 | 0,2293 | 0,2299 |
| aabb | 0,3050 | 0,3055 | 0,3061 | 0,3069 | 0,3078 |
| **Penetrance/genotype** |  |  |  |  |  |
| **Cumulative** |  |  |  |  |  |
| AABB | 0,6580 | 0,5883 | 0,4962 | 0,3745 | 0,2134 |
| AABb | 0,9120 | 0,8154 | 0,6877 | 0,5190 | 0,2958 |
| AAbb | 0,9250 | 0,8270 | 0,6975 | 0,5264 | 0,3000 |
| AaBB | 0,9330 | 0,8341 | 0,7036 | 0,5310 | 0,3026 |
| AaBb | 0,4310 | 0,3853 | 0,3250 | 0,2453 | 0,1398 |
| Aabb | 0,3750 | 0,3353 | 0,2828 | 0,2134 | 0,1216 |
| aaBB | 0,7580 | 0,6777 | 0,5716 | 0,4314 | 0,2458 |
| aaBb | 0,7090 | 0,6339 | 0,5346 | 0,4035 | 0,2299 |
| aabb | 0,9490 | 0,8484 | 0,7156 | 0,5401 | 0,3078 |

**Model 13 & 14 – Decreasing failure rate survival distribution; H^2^(5) = 0.20; K(5) = 0.750**

|  |  | **Time-point** | | |  |
| --- | --- | --- | --- | --- | --- |
|  | **5** | **4** | **3** | **2** | **1** |
| **K** |  |  |  |  |  |
| Time-point | 0,2411 | 0,2415 | 0,2419 | 0,2425 | 0,2432 |
| Cumulative | 0,7498 | 0,6704 | 0,5654 | 0,4267 | 0,2432 |
| **H^2^** |  |  |  |  |  |
| Time-point | 0,0219 | 0,0218 | 0,0218 | 0,0218 | 0,0223 |
| Cumulative | 0,2053 | 0,1395 | 0,0892 | 0,0511 | 0,0223 |
| **Penetrance/genotype** |  |  |  |  |  |
| **Time-point** |  |  |  |  |  |
| AABB | 0,2073 | 0,2076 | 0,2080 | 0,2086 | 0,2092 |
| AABb | 0,3086 | 0,3091 | 0,3096 | 0,3104 | 0,3113 |
| AAbb | 0,2411 | 0,2415 | 0,2419 | 0,2425 | 0,2432 |
| AaBB | 0,3054 | 0,3058 | 0,3064 | 0,3072 | 0,3081 |
| AaBb | 0,1138 | 0,1140 | 0,1142 | 0,1145 | 0,1148 |
| Aabb | 0,2311 | 0,2315 | 0,2319 | 0,2325 | 0,2332 |
| aaBB | 0,2668 | 0,2672 | 0,2677 | 0,2684 | 0,2692 |
| aaBb | 0,1797 | 0,1800 | 0,1803 | 0,1808 | 0,1813 |
| aabb | 0,3205 | 0,3210 | 0,3216 | 0,3224 | 0,3233 |
| **Penetrance/genotype** |  |  |  |  |  |
| **Cumulative** |  |  |  |  |  |
| AABB | 0,6450 | 0,5766 | 0,4864 | 0,3671 | 0,2092 |
| AABb | 0,9600 | 0,8583 | 0,7239 | 0,5463 | 0,3113 |
| AAbb | 0,7500 | 0,6705 | 0,5656 | 0,4268 | 0,2432 |
| AaBB | 0,9500 | 0,8493 | 0,7164 | 0,5406 | 0,3081 |
| AaBb | 0,3540 | 0,3165 | 0,2669 | 0,2015 | 0,1148 |
| Aabb | 0,7190 | 0,6428 | 0,5422 | 0,4092 | 0,2332 |
| aaBB | 0,8300 | 0,7420 | 0,6259 | 0,4724 | 0,2692 |
| aaBb | 0,5590 | 0,4998 | 0,4215 | 0,3181 | 0,1813 |
| aabb | 0,9970 | 0,8913 | 0,7518 | 0,5674 | 0,3233 |

**Model 15 & 16 – Decreasing failure rate survival distribution; H^2^(5) = 0.25; K(5) = 0.750**

|  |  | **Time-point** | | |  |
| --- | --- | --- | --- | --- | --- |
|  | **5** | **4** | **3** | **2** | **1** |
| **K** |  |  |  |  |  |
| Time-point | 0,2411 | 0,2415 | 0,2419 | 0,2425 | 0,2432 |
| Cumulative | 0,7498 | 0,6704 | 0,5654 | 0,4267 | 0,2432 |
| **H^2^** |  |  |  |  |  |
| Time-point | 0,0262 | 0,0267 | 0,0267 | 0,0267 | 0,0266 |
| Cumulative | 0,2496 | 0,1694 | 0,1083 | 0,0621 | 0,0266 |
| **Penetrance/genotype** |  |  |  |  |  |
| **Time-point** |  |  |  |  |  |
| AABB | 0,2041 | 0,2044 | 0,2048 | 0,2053 | 0,2059 |
| AABb | 0,3153 | 0,3158 | 0,3164 | 0,3172 | 0,3181 |
| AAbb | 0,2411 | 0,2415 | 0,2419 | 0,2425 | 0,2432 |
| AaBB | 0,3121 | 0,3126 | 0,3132 | 0,3140 | 0,3149 |
| AaBb | 0,1000 | 0,1001 | 0,1003 | 0,1006 | 0,1009 |
| Aabb | 0,2311 | 0,2315 | 0,2319 | 0,2325 | 0,2332 |
| aaBB | 0,2668 | 0,2672 | 0,2677 | 0,2684 | 0,2692 |
| aaBb | 0,1797 | 0,1800 | 0,1803 | 0,1808 | 0,1813 |
| aabb | 0,3205 | 0,3210 | 0,3216 | 0,3224 | 0,3233 |
| **Penetrance/genotype** |  |  |  |  |  |
| **Cumulative** |  |  |  |  |  |
| AABB | 0,6350 | 0,5677 | 0,4788 | 0,3614 | 0,2059 |
| AABb | 0,9810 | 0,8770 | 0,7398 | 0,5583 | 0,3182 |
| AAbb | 0,7500 | 0,6705 | 0,5656 | 0,4268 | 0,2432 |
| AaBB | 0,9710 | 0,8681 | 0,7322 | 0,5526 | 0,3149 |
| AaBb | 0,3110 | 0,2780 | 0,2345 | 0,1770 | 0,1009 |
| Aabb | 0,7190 | 0,6428 | 0,5422 | 0,4092 | 0,2332 |
| aaBB | 0,8300 | 0,7420 | 0,6259 | 0,4724 | 0,2692 |
| aaBb | 0,5590 | 0,4998 | 0,4215 | 0,3181 | 0,1813 |
| aabb | 0,9970 | 0,8913 | 0,7518 | 0,5674 | 0,3233 |

**Model 17 & 18 – Increasing failure rate survival distribution; H^2^(5) = 0.10; K(5) = 0.750**

|  | **Time-Point** | | | | |
| --- | --- | --- | --- | --- | --- |
|  | **5** | **4** | **3** | **2** | **1** |
| **K** |  |  |  |  |  |
| Time-point | 0,2509 | 0,2474 | 0,2431 | 0,2381 | 0,2325 |
| Cumulative | 0,7505 | 0,6669 | 0,5574 | 0,4152 | 0,2325 |
| **H2** |  |  |  |  |  |
| Time-point | 0,0112 | 0,0107 | 0,0109 | 0,0105 | 0,0101 |
| Cumulative | 0,1003 | 0,0670 | 0,0421 | 0,0239 | 0,0101 |
| **Penetrance/genotype** |  |  |  |  |  |
| **Time-point** |  |  |  |  |  |
| AABB | 0,2261 | 0,2230 | 0,2191 | 0,2146 | 0,2095 |
| AABb | 0,2930 | 0,2889 | 0,2840 | 0,2781 | 0,2715 |
| AAbb | 0,3094 | 0,3051 | 0,2999 | 0,2937 | 0,2867 |
| AaBB | 0,3000 | 0,2959 | 0,2908 | 0,2848 | 0,2781 |
| AaBb | 0,1683 | 0,1659 | 0,1631 | 0,1597 | 0,1559 |
| Aabb | 0,1254 | 0,1237 | 0,1216 | 0,1191 | 0,1162 |
| aaBB | 0,2536 | 0,2500 | 0,2457 | 0,2407 | 0,2350 |
| aaBb | 0,2372 | 0,2339 | 0,2298 | 0,2251 | 0,2198 |
| aabb | 0,3174 | 0,3130 | 0,3076 | 0,3013 | 0,2942 |
| **Penetrance/genotype** |  |  |  |  |  |
| **Cumulative** |  |  |  |  |  |
| AABB | 0,6760 | 0,6007 | 0,5021 | 0,3740 | 0,2094 |
| AABb | 0,8760 | 0,7785 | 0,6506 | 0,4847 | 0,2714 |
| AAbb | 0,9250 | 0,8220 | 0,6870 | 0,5118 | 0,2865 |
| AaBB | 0,8970 | 0,7971 | 0,6662 | 0,4963 | 0,2779 |
| AaBb | 0,5030 | 0,4470 | 0,3736 | 0,2783 | 0,1558 |
| Aabb | 0,3750 | 0,3332 | 0,2785 | 0,2075 | 0,1162 |
| aaBB | 0,7580 | 0,6736 | 0,5630 | 0,4194 | 0,2348 |
| aaBb | 0,7090 | 0,6301 | 0,5266 | 0,3923 | 0,2196 |
| aabb | 0,9490 | 0,8433 | 0,7049 | 0,5250 | 0,2940 |

**Model 19 & 20 – Increasing failure rate survival distribution; H^2^(5) = 0.15; K(5) = 0.750**

|  |  | **Time-Point** | | |  |
| --- | --- | --- | --- | --- | --- |
|  | **5** | **4** | **3** | **2** | **1** |
| **K** |  |  |  |  |  |
| Time-point | 0,2509 | 0,2474 | 0,2431 | 0,2381 | 0,2325 |
| Cumulative | 0,7505 | 0,6669 | 0,5574 | 0,4152 | 0,2325 |
| **H^2^** |  |  |  |  |  |
| Time-point | 0,0170 | 0,0166 | 0,0163 | 0,0160 | 0,0157 |
| Cumulative | 0,1536 | 0,1020 | 0,0644 | 0,0362 | 0,0157 |
| **Penetrance/genotype** |  |  |  |  |  |
| **Time-point** |  |  |  |  |  |
| AABB | 0,2201 | 0,2170 | 0,2133 | 0,2089 | 0,2040 |
| AABb | 0,3051 | 0,3008 | 0,2956 | 0,2895 | 0,2827 |
| AAbb | 0,3094 | 0,3051 | 0,2999 | 0,2937 | 0,2867 |
| AaBB | 0,3121 | 0,3078 | 0,3025 | 0,2962 | 0,2892 |
| AaBb | 0,1442 | 0,1422 | 0,1397 | 0,1368 | 0,1336 |
| Aabb | 0,1254 | 0,1237 | 0,1216 | 0,1191 | 0,1162 |
| aaBB | 0,2536 | 0,2500 | 0,2457 | 0,2407 | 0,2350 |
| aaBb | 0,2372 | 0,2339 | 0,2298 | 0,2251 | 0,2198 |
| aabb | 0,3174 | 0,3130 | 0,3076 | 0,3013 | 0,2942 |
| **Penetrance/genotype** |  |  |  |  |  |
| **Cumulative** |  |  |  |  |  |
| AABB | 0,6580 | 0,5847 | 0,4887 | 0,3640 | 0,2038 |
| AABb | 0,9120 | 0,8104 | 0,6774 | 0,5046 | 0,2825 |
| AAbb | 0,9250 | 0,8220 | 0,6870 | 0,5118 | 0,2865 |
| AaBB | 0,9330 | 0,8291 | 0,6930 | 0,5162 | 0,2890 |
| AaBb | 0,4310 | 0,3830 | 0,3201 | 0,2385 | 0,1335 |
| Aabb | 0,3750 | 0,3332 | 0,2785 | 0,2075 | 0,1162 |
| aaBB | 0,7580 | 0,6736 | 0,5630 | 0,4194 | 0,2348 |
| aaBb | 0,7090 | 0,6301 | 0,5266 | 0,3923 | 0,2196 |
| aabb | 0,9490 | 0,8433 | 0,7049 | 0,5250 | 0,2940 |

**Model 21 & 22 – Increasing failure rate survival distribution; H^2^(5) = 0.20; K(5) = 0.750**

|  |  | **Time-Point** | | |  |
| --- | --- | --- | --- | --- | --- |
|  | **5** | **4** | **3** | **2** | **1** |
| **K** |  |  |  |  |  |
| Time-point | 0,2509 | 0,2474 | 0,2431 | 0,2381 | 0,2325 |
| Cumulative | 0,7505 | 0,6669 | 0,5574 | 0,4152 | 0,2325 |
| **H^2^** |  |  |  |  |  |
| Time-point | 0,0229 | 0,0226 | 0,0217 | 0,0215 | 0,0207 |
| Cumulative | 0,2053 | 0,1367 | 0,0863 | 0,0486 | 0,0208 |
| **Penetrance/genotype** |  |  |  |  |  |
| **Time-point** |  |  |  |  |  |
| AABB | 0,2158 | 0,2128 | 0,2091 | 0,2048 | 0,1999 |
| AABb | 0,3211 | 0,3167 | 0,3112 | 0,3048 | 0,2976 |
| AAbb | 0,2509 | 0,2474 | 0,2431 | 0,2381 | 0,2325 |
| AaBB | 0,3178 | 0,3134 | 0,3080 | 0,3016 | 0,2945 |
| AaBb | 0,1184 | 0,1168 | 0,1148 | 0,1124 | 0,1097 |
| Aabb | 0,2405 | 0,2372 | 0,2331 | 0,2283 | 0,2229 |
| aaBB | 0,2776 | 0,2738 | 0,2691 | 0,2635 | 0,2573 |
| aaBb | 0,1870 | 0,1844 | 0,1812 | 0,1775 | 0,1733 |
| aabb | 0,3335 | 0,3289 | 0,3232 | 0,3165 | 0,3091 |
| **Penetrance/genotype** |  |  |  |  |  |
| **Cumulative** |  |  |  |  |  |
| AABB | 0,6450 | 0,5732 | 0,4791 | 0,3569 | 0,1998 |
| AABb | 0,9600 | 0,8531 | 0,7130 | 0,5311 | 0,2974 |
| AAbb | 0,7500 | 0,6665 | 0,5570 | 0,4149 | 0,2323 |
| AaBB | 0,9500 | 0,8442 | 0,7056 | 0,5256 | 0,2943 |
| AaBb | 0,3540 | 0,3146 | 0,2629 | 0,1959 | 0,1097 |
| Aabb | 0,7190 | 0,6389 | 0,5340 | 0,3978 | 0,2227 |
| aaBB | 0,8300 | 0,7376 | 0,6165 | 0,4592 | 0,2571 |
| aaBb | 0,5590 | 0,4968 | 0,4152 | 0,3093 | 0,1732 |
| aabb | 0,9970 | 0,8860 | 0,7405 | 0,5516 | 0,3088 |

**Model 23 & 24 – Increasing failure rate survival distribution; H^2^(5) = 0.25; K(5) = 0.750**

|  |  | **Time-Point** | | |  |
| --- | --- | --- | --- | --- | --- |
|  | **5** | **4** | **3** | **2** | **1** |
| **K** |  |  |  |  |  |
| Time-point | 0,2509 | 0,2474 | 0,2431 | 0,2381 | 0,2325 |
| Cumulative | 0,7505 | 0,6669 | 0,5574 | 0,4152 | 0,2325 |
| **H^2^** |  |  |  |  |  |
| Time-point | 0,0277 | 0,0274 | 0,0266 | 0,0259 | 0,0252 |
| Cumulative | 0,2496 | 0,1665 | 0,1046 | 0,0589 | 0,0253 |
| **Penetrance/genotype** |  |  |  |  |  |
| **Time-point** |  |  |  |  |  |
| AABB | 0,2124 | 0,2095 | 0,2059 | 0,2016 | 0,1968 |
| AABb | 0,3281 | 0,3236 | 0,3180 | 0,3115 | 0,3041 |
| AAbb | 0,2509 | 0,2474 | 0,2431 | 0,2381 | 0,2325 |
| AaBB | 0,3248 | 0,3203 | 0,3148 | 0,3083 | 0,3010 |
| AaBb | 0,1040 | 0,1026 | 0,1008 | 0,0987 | 0,0964 |
| Aabb | 0,2405 | 0,2372 | 0,2331 | 0,2283 | 0,2229 |
| aaBB | 0,2776 | 0,2738 | 0,2691 | 0,2635 | 0,2573 |
| aaBb | 0,1870 | 0,1844 | 0,1812 | 0,1775 | 0,1733 |
| aabb | 0,3335 | 0,3289 | 0,3232 | 0,3165 | 0,3091 |
| **Penetrance/genotype** |  |  |  |  |  |
| **Cumulative** |  |  |  |  |  |
| AABB | 0,6350 | 0,5643 | 0,4716 | 0,3513 | 0,1967 |
| AABb | 0,9810 | 0,8718 | 0,7286 | 0,5428 | 0,3039 |
| AAbb | 0,7500 | 0,6665 | 0,5570 | 0,4149 | 0,2323 |
| AaBB | 0,9710 | 0,8629 | 0,7212 | 0,5372 | 0,3008 |
| AaBb | 0,3110 | 0,2764 | 0,2310 | 0,1721 | 0,0963 |
| Aabb | 0,7190 | 0,6389 | 0,5340 | 0,3978 | 0,2227 |
| aaBB | 0,8300 | 0,7376 | 0,6165 | 0,4592 | 0,2571 |
| aaBb | 0,5590 | 0,4968 | 0,4152 | 0,3093 | 0,1732 |
| aabb | 0,9970 | 0,8860 | 0,7405 | 0,5516 | 0,3088 |

**Model 25 & 26 – Bathtub-shaped rate survival distribution; H^2^(5) = 0.10; K(5) = 0.750**

|  | **Time-Point** | | | | |
| --- | --- | --- | --- | --- | --- |
|  | **5** | **4** | **3** | **2** | **1** |
| **K** |  |  |  |  |  |
| Time-point | 0,2441 | 0,2381 | 0,2337 | 0,2355 | 0,2590 |
| Cumulative | 0,7500 | 0,6692 | 0,5659 | 0,4335 | 0,2590 |
| **H2** |  |  |  |  |  |
| Time-point | 0,0108 | 0,0105 | 0,0101 | 0,0106 | 0,0115 |
| Cumulative | 0,1003 | 0,0677 | 0,0436 | 0,0256 | 0,0115 |
| **Penetrance/genotype** |  |  |  |  |  |
| **Time-point** |  |  |  |  |  |
| AABB | 0,2201 | 0,2146 | 0,2106 | 0,2122 | 0,2334 |
| AABb | 0,2852 | 0,2781 | 0,2729 | 0,2750 | 0,3025 |
| AAbb | 0,3011 | 0,2936 | 0,2882 | 0,2904 | 0,3194 |
| AaBB | 0,2920 | 0,2848 | 0,2795 | 0,2816 | 0,3098 |
| AaBb | 0,1637 | 0,1597 | 0,1567 | 0,1579 | 0,1737 |
| Aabb | 0,1221 | 0,1190 | 0,1168 | 0,1177 | 0,1295 |
| aaBB | 0,2467 | 0,2406 | 0,2362 | 0,2380 | 0,2618 |
| aaBb | 0,2308 | 0,2251 | 0,2209 | 0,2226 | 0,2448 |
| aabb | 0,3089 | 0,3013 | 0,2957 | 0,2980 | 0,3277 |
| **Penetrance/genotype** |  |  |  |  |  |
| **Cumulative** |  |  |  |  |  |
| AABB | 0,6760 | 0,6032 | 0,5101 | 0,3908 | 0,2335 |
| AABb | 0,8760 | 0,7817 | 0,6610 | 0,5064 | 0,3026 |
| AAbb | 0,9250 | 0,8254 | 0,6980 | 0,5347 | 0,3195 |
| AaBB | 0,8970 | 0,8004 | 0,6768 | 0,5185 | 0,3098 |
| AaBb | 0,5030 | 0,4488 | 0,3795 | 0,2908 | 0,1737 |
| Aabb | 0,3750 | 0,3346 | 0,2830 | 0,2168 | 0,1295 |
| aaBB | 0,7580 | 0,6764 | 0,5719 | 0,4382 | 0,2618 |
| aaBb | 0,7090 | 0,6327 | 0,5350 | 0,4098 | 0,2449 |
| aabb | 0,9490 | 0,8468 | 0,7161 | 0,5486 | 0,3278 |

**Model 27 & 28 – Bathtub-shaped rate survival distribution; H^2^(5) = 0.15; K(5) = 0.750**

|  |  | **Time-point** | | |  |
| --- | --- | --- | --- | --- | --- |
|  | **5** | **4** | **3** | **2** | **1** |
| **K** |  |  |  |  |  |
| Time-point | 0,2441 | 0,2381 | 0,2337 | 0,2355 | 0,2590 |
| Cumulative | 0,7500 | 0,6692 | 0,5659 | 0,4335 | 0,2590 |
| **H^2^** |  |  |  |  |  |
| Time-point | 0,0168 | 0,0160 | 0,0156 | 0,0156 | 0,0177 |
| Cumulative | 0,1536 | 0,1034 | 0,0668 | 0,0391 | 0,0177 |
| **Penetrance/genotype** |  |  |  |  |  |
| **Time-point** |  |  |  |  |  |
| AABB | 0,2142 | 0,2089 | 0,2050 | 0,2066 | 0,2272 |
| AABb | 0,2969 | 0,2895 | 0,2841 | 0,2863 | 0,3149 |
| AAbb | 0,3011 | 0,2936 | 0,2882 | 0,2904 | 0,3194 |
| AaBB | 0,3037 | 0,2962 | 0,2907 | 0,2929 | 0,3222 |
| AaBb | 0,1403 | 0,1368 | 0,1343 | 0,1353 | 0,1488 |
| Aabb | 0,1221 | 0,1190 | 0,1168 | 0,1177 | 0,1295 |
| aaBB | 0,2467 | 0,2406 | 0,2362 | 0,2380 | 0,2618 |
| aaBb | 0,2308 | 0,2251 | 0,2209 | 0,2226 | 0,2448 |
| aabb | 0,3089 | 0,3013 | 0,2957 | 0,2980 | 0,3277 |
| **Penetrance/genotype** |  |  |  |  |  |
| **Cumulative** |  |  |  |  |  |
| AABB | 0,6580 | 0,5872 | 0,4965 | 0,3804 | 0,2273 |
| AABb | 0,9120 | 0,8138 | 0,6881 | 0,5272 | 0,3150 |
| AAbb | 0,9250 | 0,8254 | 0,6980 | 0,5347 | 0,3195 |
| AaBB | 0,9330 | 0,8325 | 0,7040 | 0,5393 | 0,3222 |
| AaBb | 0,4310 | 0,3846 | 0,3252 | 0,2491 | 0,1489 |
| Aabb | 0,3750 | 0,3346 | 0,2830 | 0,2168 | 0,1295 |
| aaBB | 0,7580 | 0,6764 | 0,5719 | 0,4382 | 0,2618 |
| aaBb | 0,7090 | 0,6327 | 0,5350 | 0,4098 | 0,2449 |
| aabb | 0,9490 | 0,8468 | 0,7161 | 0,5486 | 0,3278 |

**Model 29 & 30 – Bathtub-shaped rate survival distribution; H^2^(5) = 0.20; K(5) = 0.750**

|  |  | **Time-point** | | |  |
| --- | --- | --- | --- | --- | --- |
|  | **5** | **4** | **3** | **2** | **1** |
| **K** |  |  |  |  |  |
| Time-point | 0,2441 | 0,2381 | 0,2337 | 0,2355 | 0,2590 |
| Cumulative | 0,7500 | 0,6692 | 0,5659 | 0,4335 | 0,2590 |
| **H^2^** |  |  |  |  |  |
| Time-point | 0,0222 | 0,0215 | 0,0207 | 0,0211 | 0,0240 |
| Cumulative | 0,2053 | 0,1386 | 0,0892 | 0,0525 | 0,0240 |
| **Penetrance/genotype** |  |  |  |  |  |
| **Time-point** |  |  |  |  |  |
| AABB | 0,2100 | 0,2048 | 0,2009 | 0,2025 | 0,2227 |
| AABb | 0,3125 | 0,3048 | 0,2991 | 0,3014 | 0,3315 |
| AAbb | 0,2441 | 0,2381 | 0,2337 | 0,2355 | 0,2590 |
| AaBB | 0,3092 | 0,3016 | 0,2960 | 0,2983 | 0,3281 |
| AaBb | 0,1152 | 0,1124 | 0,1103 | 0,1111 | 0,1222 |
| Aabb | 0,2340 | 0,2282 | 0,2240 | 0,2257 | 0,2483 |
| aaBB | 0,2702 | 0,2635 | 0,2586 | 0,2606 | 0,2866 |
| aaBb | 0,1820 | 0,1775 | 0,1742 | 0,1755 | 0,1930 |
| aabb | 0,3245 | 0,3165 | 0,3106 | 0,3130 | 0,3443 |
| **Penetrance/genotype** |  |  |  |  |  |
| **Cumulative** |  |  |  |  |  |
| AABB | 0,6450 | 0,5756 | 0,4867 | 0,3728 | 0,2228 |
| AABb | 0,9600 | 0,8566 | 0,7244 | 0,5549 | 0,3316 |
| AAbb | 0,7500 | 0,6692 | 0,5659 | 0,4335 | 0,2590 |
| AaBB | 0,9500 | 0,8477 | 0,7168 | 0,5492 | 0,3281 |
| AaBb | 0,3540 | 0,3159 | 0,2671 | 0,2046 | 0,1223 |
| Aabb | 0,7190 | 0,6416 | 0,5425 | 0,4156 | 0,2483 |
| aaBB | 0,8300 | 0,7406 | 0,6263 | 0,4798 | 0,2867 |
| aaBb | 0,5590 | 0,4988 | 0,4218 | 0,3231 | 0,1931 |
| aabb | 0,9970 | 0,8897 | 0,7523 | 0,5763 | 0,3443 |

**Model 31 & 32 – Bathtub-shaped rate survival distribution; H^2^(5) = 0.25; K(5) = 0.750**

|  |  | **Time-point** | | |  |
| --- | --- | --- | --- | --- | --- |
|  | **5** | **4** | **3** | **2** | **1** |
| **K** |  |  |  |  |  |
| Time-point | 0,2441 | 0,2381 | 0,2337 | 0,2355 | 0,2590 |
| Cumulative | 0,7500 | 0,6692 | 0,5659 | 0,4335 | 0,2590 |
| **H^2^** |  |  |  |  |  |
| Time-point | 0,0271 | 0,0259 | 0,0251 | 0,0255 | 0,0292 |
| Cumulative | 0,2496 | 0,1687 | 0,1087 | 0,0635 | 0,0292 |
| **Penetrance/genotype** |  |  |  |  |  |
| **Time-point** |  |  |  |  |  |
| AABB | 0,2067 | 0,2016 | 0,1978 | 0,1994 | 0,2193 |
| AABb | 0,3193 | 0,3114 | 0,3056 | 0,3080 | 0,3388 |
| AAbb | 0,2441 | 0,2381 | 0,2337 | 0,2355 | 0,2590 |
| AaBB | 0,3161 | 0,3082 | 0,3025 | 0,3049 | 0,3353 |
| AaBb | 0,1012 | 0,0987 | 0,0969 | 0,0976 | 0,1074 |
| Aabb | 0,2340 | 0,2282 | 0,2240 | 0,2257 | 0,2483 |
| aaBB | 0,2702 | 0,2635 | 0,2586 | 0,2606 | 0,2866 |
| aaBb | 0,1820 | 0,1775 | 0,1742 | 0,1755 | 0,1930 |
| aabb | 0,3245 | 0,3165 | 0,3106 | 0,3130 | 0,3443 |
| **Penetrance/genotype** |  |  |  |  |  |
| **Cumulative** |  |  |  |  |  |
| AABB | 0,6350 | 0,5666 | 0,4791 | 0,3671 | 0,2193 |
| AABb | 0,9810 | 0,8754 | 0,7402 | 0,5671 | 0,3388 |
| AAbb | 0,7500 | 0,6692 | 0,5659 | 0,4335 | 0,2590 |
| AaBB | 0,9710 | 0,8665 | 0,7327 | 0,5613 | 0,3354 |
| AaBb | 0,3110 | 0,2775 | 0,2347 | 0,1798 | 0,1074 |
| Aabb | 0,7190 | 0,6416 | 0,5425 | 0,4156 | 0,2483 |
| aaBB | 0,8300 | 0,7406 | 0,6263 | 0,4798 | 0,2867 |
| aaBb | 0,5590 | 0,4988 | 0,4218 | 0,3231 | 0,1931 |
| aabb | 0,9970 | 0,8897 | 0,7523 | 0,5763 | 0,3443 |

**Model 33 & 34 –Exponential survival distribution; H^2^(5) = 0.10; K(5) = 0.750**

|  | **Time-Point** | | | | |
| --- | --- | --- | --- | --- | --- |
|  | **5** | **4** | **3** | **2** | **1** |
| **K** |  |  |  |  |  |
| Time-point | 0,2419 | 0,2420 | 0,2419 | 0,2420 | 0,2419 |
| Cumulative | 0,7497 | 0,6698 | 0,5644 | 0,4254 | 0,2419 |
| **H2** |  |  |  |  |  |
| Time-point | 0,0109 | 0,0109 | 0,0109 | 0,0109 | 0,0109 |
| Cumulative | 0,1003 | 0,0678 | 0,0435 | 0,0249 | 0,0109 |
| **Penetrance/genotype** |  |  |  |  |  |
| **Time-point** |  |  |  |  |  |
| AABB | 0,2181 | 0,2181 | 0,2181 | 0,2181 | 0,2181 |
| AABb | 0,2826 | 0,2826 | 0,2826 | 0,2826 | 0,2826 |
| AAbb | 0,2984 | 0,2985 | 0,2984 | 0,2984 | 0,2984 |
| AaBB | 0,2894 | 0,2894 | 0,2894 | 0,2894 | 0,2893 |
| AaBb | 0,1623 | 0,1623 | 0,1623 | 0,1623 | 0,1622 |
| Aabb | 0,1210 | 0,1210 | 0,1210 | 0,1210 | 0,1210 |
| aaBB | 0,2445 | 0,2446 | 0,2445 | 0,2446 | 0,2445 |
| aaBb | 0,2287 | 0,2288 | 0,2287 | 0,2288 | 0,2287 |
| aabb | 0,3061 | 0,3062 | 0,3061 | 0,3062 | 0,3061 |
| **Penetrance/genotype** |  |  |  |  |  |
| **Cumulative** |  |  |  |  |  |
| AABB | 0,6760 | 0,6040 | 0,5089 | 0,3836 | 0,2182 |
| AABb | 0,8760 | 0,7826 | 0,6595 | 0,4970 | 0,2827 |
| AAbb | 0,9250 | 0,8264 | 0,6964 | 0,5248 | 0,2985 |
| AaBB | 0,8970 | 0,8014 | 0,6753 | 0,5089 | 0,2895 |
| AaBb | 0,5030 | 0,4494 | 0,3787 | 0,2854 | 0,1623 |
| Aabb | 0,3750 | 0,3350 | 0,2823 | 0,2128 | 0,1210 |
| aaBB | 0,7580 | 0,6772 | 0,5707 | 0,4301 | 0,2446 |
| aaBb | 0,7090 | 0,6334 | 0,5338 | 0,4023 | 0,2288 |
| aabb | 0,9490 | 0,8479 | 0,7144 | 0,5384 | 0,3063 |

**Model 35 & 36 –Exponential survival distribution; H^2^(5) = 0.15; K(5) = 0.750**

|  |  | **Time-point** | | |  |
| --- | --- | --- | --- | --- | --- |
|  | **5** | **4** | **3** | **2** | **1** |
| **K** |  |  |  |  |  |
| Time-point | 0,2423 | 0,2423 | 0,2424 | 0,2423 | 0,2423 |
| Cumulative | 0,7503 | 0,6704 | 0,5650 | 0,4259 | 0,2423 |
| **H^2^** |  |  |  |  |  |
| Time-point | 0,0163 | 0,0163 | 0,0163 | 0,0163 | 0,0163 |
| Cumulative | 0,1536 | 0,1040 | 0,0663 | 0,0380 | 0,0164 |
| **Penetrance/genotype** |  |  |  |  |  |
| **Time-point** |  |  |  |  |  |
| AABB | 0,2126 | 0,2126 | 0,2126 | 0,2126 | 0,2126 |
| AABb | 0,2947 | 0,2947 | 0,2947 | 0,2946 | 0,2947 |
| AAbb | 0,2989 | 0,2989 | 0,2989 | 0,2988 | 0,2989 |
| AaBB | 0,3015 | 0,3015 | 0,3015 | 0,3014 | 0,3014 |
| AaBb | 0,1393 | 0,1393 | 0,1393 | 0,1392 | 0,1393 |
| Aabb | 0,1212 | 0,1212 | 0,1212 | 0,1211 | 0,1212 |
| aaBB | 0,2449 | 0,2449 | 0,2450 | 0,2449 | 0,2449 |
| aaBb | 0,2291 | 0,2291 | 0,2291 | 0,2291 | 0,2291 |
| aabb | 0,3066 | 0,3067 | 0,3067 | 0,3066 | 0,3066 |
| **Penetrance/genotype** |  |  |  |  |  |
| **Cumulative** |  |  |  |  |  |
| AABB | 0,6580 | 0,5880 | 0,4955 | 0,3735 | 0,2125 |
| AABb | 0,9120 | 0,8149 | 0,6868 | 0,5177 | 0,2945 |
| AAbb | 0,9250 | 0,8265 | 0,6966 | 0,5251 | 0,2987 |
| AaBB | 0,9330 | 0,8337 | 0,7026 | 0,5296 | 0,3013 |
| AaBb | 0,4310 | 0,3851 | 0,3246 | 0,2447 | 0,1392 |
| Aabb | 0,3750 | 0,3351 | 0,2824 | 0,2129 | 0,1211 |
| aaBB | 0,7580 | 0,6773 | 0,5708 | 0,4303 | 0,2448 |
| aaBb | 0,7090 | 0,6335 | 0,5339 | 0,4025 | 0,2290 |
| aabb | 0,9490 | 0,8480 | 0,7147 | 0,5387 | 0,3065 |

**Model 37 & 38 – Exponential survival distribution; H^2^(5) = 0.20; K(5) = 0.750**

|  |  | **Time-point** | | |  |
| --- | --- | --- | --- | --- | --- |
|  | **5** | **4** | **3** | **2** | **1** |
| **K** |  |  |  |  |  |
| Time-point | 0,2423 | 0,2423 | 0,2424 | 0,2423 | 0,2423 |
| Cumulative | 0,7503 | 0,6704 | 0,5650 | 0,4259 | 0,2423 |
| **H^2^** |  |  |  |  |  |
| Time-point | 0,0218 | 0,0218 | 0,0218 | 0,0218 | 0,0218 |
| Cumulative | 0,2053 | 0,1393 | 0,0891 | 0,0507 | 0,0218 |
| **Penetrance/genotype** |  |  |  |  |  |
| **Time-point** |  |  |  |  |  |
| AABB | 0,2084 | 0,2084 | 0,2084 | 0,2084 | 0,2084 |
| AABb | 0,3102 | 0,3102 | 0,3102 | 0,3101 | 0,3102 |
| AAbb | 0,2423 | 0,2423 | 0,2424 | 0,2423 | 0,2423 |
| AaBB | 0,3069 | 0,3070 | 0,3070 | 0,3069 | 0,3069 |
| AaBb | 0,1144 | 0,1144 | 0,1144 | 0,1144 | 0,1144 |
| Aabb | 0,2323 | 0,2323 | 0,2324 | 0,2323 | 0,2323 |
| aaBB | 0,2682 | 0,2682 | 0,2682 | 0,2681 | 0,2682 |
| aaBb | 0,1806 | 0,1806 | 0,1806 | 0,1806 | 0,1806 |
| aabb | 0,3221 | 0,3222 | 0,3222 | 0,3221 | 0,3221 |
| **Penetrance/genotype** |  |  |  |  |  |
| **Cumulative** |  |  |  |  |  |
| AABB | 0,6450 | 0,5763 | 0,4857 | 0,3662 | 0,2083 |
| AABb | 0,9600 | 0,8578 | 0,7230 | 0,5450 | 0,3101 |
| AAbb | 0,7500 | 0,6702 | 0,5648 | 0,4258 | 0,2422 |
| AaBB | 0,9500 | 0,8489 | 0,7154 | 0,5393 | 0,3068 |
| AaBb | 0,3540 | 0,3163 | 0,2666 | 0,2010 | 0,1143 |
| Aabb | 0,7190 | 0,6425 | 0,5415 | 0,4082 | 0,2322 |
| aaBB | 0,8300 | 0,7417 | 0,6251 | 0,4712 | 0,2681 |
| aaBb | 0,5590 | 0,4995 | 0,4210 | 0,3173 | 0,1805 |
| aabb | 0,9970 | 0,8909 | 0,7508 | 0,5660 | 0,3220 |

**Model 39 & 40 – Exponential survival distribution; H^2^(5) = 0.25; K(5) = 0.750**

|  |  | **Time-point** | | |  |
| --- | --- | --- | --- | --- | --- |
|  | **5** | **4** | **3** | **2** | **1** |
| **K** |  |  |  |  |  |
| Time-point | 0,2423 | 0,2424 | 0,2423 | 0,2423 | 0,2423 |
| Cumulative | 0,7503 | 0,6704 | 0,5650 | 0,4259 | 0,2423 |
| **H^2^** |  |  |  |  |  |
| Time-point | 0,0267 | 0,0267 | 0,0267 | 0,0267 | 0,0267 |
| Cumulative | 0,2496 | 0,1692 | 0,1082 | 0,0618 | 0,0267 |
| **Penetrance/genotype** |  |  |  |  |  |
| **Time-point** |  |  |  |  |  |
| AABB | 0,2052 | 0,2052 | 0,2051 | 0,2051 | 0,2052 |
| AABb | 0,3170 | 0,3170 | 0,3169 | 0,3169 | 0,3170 |
| AAbb | 0,2423 | 0,2423 | 0,2423 | 0,2423 | 0,2423 |
| AaBB | 0,3137 | 0,3138 | 0,3137 | 0,3137 | 0,3137 |
| AaBb | 0,1005 | 0,1005 | 0,1005 | 0,1005 | 0,1005 |
| Aabb | 0,2323 | 0,2323 | 0,2323 | 0,2323 | 0,2323 |
| aaBB | 0,2682 | 0,2682 | 0,2681 | 0,2681 | 0,2682 |
| aaBb | 0,1806 | 0,1806 | 0,1806 | 0,1806 | 0,1806 |
| aabb | 0,3221 | 0,3222 | 0,3221 | 0,3221 | 0,3221 |
| **Penetrance/genotype** |  |  |  |  |  |
| **Cumulative** |  |  |  |  |  |
| AABB | 0,6350 | 0,5674 | 0,4782 | 0,3605 | 0,2051 |
| AABb | 0,9810 | 0,8766 | 0,7388 | 0,5569 | 0,3168 |
| AAbb | 0,7500 | 0,6702 | 0,5648 | 0,4258 | 0,2422 |
| AaBB | 0,9710 | 0,8676 | 0,7312 | 0,5512 | 0,3136 |
| AaBb | 0,3110 | 0,2779 | 0,2342 | 0,1765 | 0,1004 |
| Aabb | 0,7190 | 0,6425 | 0,5415 | 0,4082 | 0,2322 |
| aaBB | 0,8300 | 0,7417 | 0,6251 | 0,4712 | 0,2681 |
| aaBb | 0,5590 | 0,4995 | 0,4210 | 0,3173 | 0,1805 |
| aabb | 0,9970 | 0,8909 | 0,7508 | 0,5660 | 0,3220 |
