# Supplementary material for: Survival dimensionality reduction (SDR): development and clinical application of an innovative approach to detect epistasis in presence of right-censored data
Source: BMC Bioinformatics. 2010 Aug 6;11:416. doi: 10.1186/1471-2105-11-416 (PMC2928804; doi:10.1186/1471-2105-11-416)
Supplement: Additional file 2 — Genotype frequencies and univariate analysis in the rheumatoid arthritis (RA) dataset. The file lists in tabular form the single nucleotide polymorphisms (SNPs) included in the RA case-control study. It also reports the associations by the log-rank test statistics under the dominant and recessive model between the studied SNPs and the occurrence of clinical remission after therapy with anti-TNF agents. [file 1471-2105-11-416-S2.DOC]

**Table S1 – Studied genes and genotype frequencies**

| **ID** | **Gene,**  **genotype** | **N** | **%** |
| --- | --- | --- | --- |
| **rs20541** | **IL-13**  CC  CT  TT | 225  112  19 | 66.1  29  4.9 |
| **rs1800925** | **IL-13**  CC  CT  TT | 258  115  13 | 66.8  29.8  3.4 |
| **rs2243250** | **IL-13**  CC  CT  TT | 272  104  10 | 70.5  26.9  2.6 |
| **rs1805010** | **IL-4R**  AA  AG  GG | 110  193  83 | 28.5  50  21.5 |
| **rs1801275** | **IL-4R**  AA  AG  GG | 240  133  13 | 62.2  34.5  3.4 |
| **rs6646259** | **IL-13R1**  AA  AG  GG | 218  110  58 | 56.5  28.5  15 |
| **rs1800629** | **TNF**  AA  AG  GG | 271  98  17 | 70.2  25.4  4.4 |
| **rs1801274** | **FcγRIIa**  CC  CT  TT | 90  244  52 | 23.3  63.2  13.5 |
| **rs396991** | **FcγRIIIa**  GG  GT  TT | 51  196  139 | 13.2  50.8  36 |
| **rs3087456** | **MHC2TA**  AA  AG  GG | 223  149  14 | 57.8  38.6  3.6 |
| **rs1061622** | **TNFRSF1b**  GG  GT  TT | 231  135  20 | 59.8  35  5.2 |
| **rs2004640** | **IRF5**  GG  GT  TT | 79  201  106 | 20.5  52.1  27.5 |
| **rs10954213** | **IRF5**  AA  AG  GG | 150  183  53 | 38.9  47.4  13.7 |
| **rs3890745** | **MMEL1/TNFRSF14**  AA  AG  GG | 183  164  39 | 47.4  42.5  10.1 |
| **rs2476601** | **PTPN22**  AA  AG  GG | 253  124  9 | 65.5  32.1  2.3 |
| **rs2816316** | **RGS1**  GG  GT  TT | 252  123  11 | 65.3  31.9  2.8 |
|  |  |  |  |
|  |  |  |  |
| **‘cont (1)** | | | |
| **ID** | **Gene,**  **N of mutant alleles** | **N** | **%** |
| **rs842647** | **REL**  AA  AG  GG | 177  170  39 | 45.9  44  10.1 |
| **rs917997** | **IL18RAP**  AA  AG  GG | 211  153  22 | 54.7  39.6  5.7 |
| **rs7574865** | **STAT4**  GG  GT  TT | 231  136  19 | 59.8  35.2  4.9 |
| **rs6441961** | **CCR1-3**  CC  CT  TT | 183  165  38 | 47.4  42.7  9.8 |
| **rs17810546** | **IL12A, SCHIP**  AA  AG  GG | 309  72  5 | 80.1  18.7  1.3 |
| **rs9811792** | **IL12A**  CC  CT  TT | 126  197  63 | 32.6  51.0  16.3 |
| **rs1464510** | **LPP**  GG  GT  TT | 114  190  82 | 29.5  49.2  21.2 |
| **rs6822844** | **IL2-IL21**  GG  GT  TT | 300  77  9 | 77.7  19.9  2.3 |
| **rs2327832** | **TNFAIP3, OLIG3**  AA  AG  GG | 254  114  18 | 65.8  29.5  4.7 |
| **rs10499194** | **TNFAIP3, OLIG3**  CC  CT  TT | 209  150  27 | 54.1  38.9  7 |
| **rs1738074** | **TAGAP**  AA  AG  GG | 135  193  58 | 35  50  15 |
| **rs42041** | **CDK6**  CC  CG  GG | 222  146  18 | 57.5  37.8  4.7 |
| **rs2812378** | **CCL21**  CC  CT  TT | 138  202  46 | 35.8  52.3  11.9 |
| **rs3761847** | **TRAF1, C5**  AA  AG  GG | 106  216  64 | 27.5  56  16.6 |
| **rs4750316** | **PFKFB3, PRKCQ**  CC  CG  GG | 241  133  12 | 62.4  34.5  3.1 |
| **rs1678542** | **KIF5A, PIP4K2C**  CC  CG  GG | 164  185  37 | 42.5  47.9  9.6 |
| **rs3184504** | **SH2B3**  CC  CT  TT | 112  182  92 | 29  47.2  23.8 |
| **‘cont (2)** | | | |
| **ID** | **Gene,**  **N of mutant alleles** | **N** | **%** |
| **rs4810485** | **CD40**  GG  GT  TT | 226  146  14 | 58.5  37.8  3.6 |
| **rs3218253** | **IL2RB**  CC  CT  TT | 190  161  35 | 49.2  41.7  9.1 |
| **rs12252317** | **EGR2**  CC  CT  TT | 326  55  5 | 84.5  14.2  1.3 |
| **rs9770242** | **PBEF1001**  AA  AC  CC | 215  145  26 | 55.7  37.6  6.7 |
| **Na** | **PBEF1543**  CC  CT TT | 213  144  29 | 55.2  37.3  7.5 |
| **rs16986050** | **CD89**  AA  AG  GG | 283  92  11 | 73.3  23.8  2.8 |

**Table S2 – Survival analysis for the rheumatoid arthritis dataset**

|  |  |  | **All Cases, DAS28 ≤ 2.6** | | | | | | |  |  |  |  |  | **Ig anti-TNF, DAS28 ≤ 2.6** | | | | | |  |  |  |
| --- | --- | --- | --- | --- | --- | --- | --- | --- | --- | --- | --- | --- | --- | --- | --- | --- | --- | --- | --- | --- | --- | --- | --- |
|  |  | **Dominant Model** | | |  |  |  | **Recessive Model** | | |  |  |  | **Dominant Model** | | |  |  |  | **Recessive Model** | | |  |
| **SNP** | **Yes (n)** | **Yes (%)** | **No (n)** | **No (%)** | **p** |  | **Yes (n)** | **Yes (%)** | **No (n)** | **No (%)** | **p** |  | **Yes (n)** | **Yes (%)** | **No (n)** | **No (%)** | **p** |  | **Yes (n)** | **Yes (%)** | **No (n)** | **No (%)** | **p** |
| **rs20541** |  |  |  |  |  |  |  |  |  |  |  |  |  |  |  |  |  |  |  |  |  |  |  |
| Cases | 113 | 91,9 | 254 | 96,6 | 0,064 |  | 50 | 40,7 | 81 | 30,8 | 0,08 |  | 103 | 92 | 228 | 97,4 | **0,044** |  | 48 | 42,9 | 70 | 29,9 | **0,028** |
| Controls | 10 | 8,1 | 9 | 3,4 |  |  | 73 | 59,3 | 182 | 69,2 |  |  | 9 | 8 | 6 | 2,6 |  |  | 64 | 57,1 | 164 | 70,1 |  |
|  |  |  |  |  |  |  |  |  |  |  |  |  |  |  |  |  |  |  |  |  |  |  |  |
| **rs1800925** |  |  |  |  |  |  |  |  |  |  |  |  |  |  |  |  |  |  |  |  |  |  |  |
| Cases | 119 | 96,7 | 254 | 96,6 | 0,992 |  | 47 | 38,2 | 81 | 30,8 | 0,157 |  | 108 | 96,4 | 226 | 96,6 | 0,954 |  | 43 | 38,4 | 71 | 30,3 | 0,159 |
| Controls | 4 | 3,3 | 9 | 3,4 |  |  | 76 | 61,8 | 182 | 69,2 |  |  | 4 | 3,6 | 8 | 3,4 |  |  | 69 | 61,6 | 163 | 69,7 |  |
|  |  |  |  |  |  |  |  |  |  |  |  |  |  |  |  |  |  |  |  |  |  |  |  |
| **rs2243250** |  |  |  |  |  |  |  |  |  |  |  |  |  |  |  |  |  |  |  |  |  |  |  |
| Cases | 119 | 96,7 | 257 | 97,7 | 0,532 |  | 41 | 33,3 | 73 | 27,8 | 0,342 |  | 109 | 97,3 | 230 | 98,3 | 0,653 |  | 39 | 34,8 | 63 | 26,9 | 0,203 |
| Controls | 4 | 3,3 | 6 | 2,3 |  |  | 82 | 66,7 | 190 | 72,2 |  |  | 3 | 2,7 | 4 | 1,7 |  |  | 73 | 65,2 | 171 | 73,1 |  |
|  |  |  |  |  |  |  |  |  |  |  |  |  |  |  |  |  |  |  |  |  |  |  |  |
| **rs1805010** |  |  |  |  |  |  |  |  |  |  |  |  |  |  |  |  |  |  |  |  |  |  |  |
| Cases | 99 | 80,5 | 204 | 77,6 | 0,451 |  | 92 | 74,8 | 184 | 70 | 0,283 |  | 89 | 79,5 | 180 | 76,9 | 0,574 |  | 86 | 76,8 | 165 | 70,5 | 0,188 |
| Controls | 24 | 19,5 | 59 | 22,4 |  |  | 31 | 25,2 | 79 | 30 |  |  | 23 | 20,5 | 54 | 23,1 |  |  | 26 | 23,2 | 69 | 29,5 |  |
|  |  |  |  |  |  |  |  |  |  |  |  |  |  |  |  |  |  |  |  |  |  |  |  |
| **rs1801275** |  |  |  |  |  |  |  |  |  |  |  |  |  |  |  |  |  |  |  |  |  |  |  |
| Cases | 120 | 97,6 | 253 | 96,2 | 0,506 |  | 40 | 32,5 | 106 | 40,3 | 0,08 |  | 110 | 98,2 | 226 | 96,6 | 0,386 |  | 34 | 30,4 | 95 | 40,6 | 0,051 |
| Controls | 3 | 2,4 | 10 | 3,8 |  |  | 83 | 67,5 | 157 | 59,7 |  |  | 2 | 1,8 | 8 | 3,4 |  |  | 78 | 69,6 | 139 | 59,4 |  |
|  |  |  |  |  |  |  |  |  |  |  |  |  |  |  |  |  |  |  |  |  |  |  |  |
| **rs6646259** |  |  |  |  |  |  |  |  |  |  |  |  |  |  |  |  |  |  |  |  |  |  |  |
| Cases | 102 | 82,9 | 226 | 85,9 | 0,42 |  | 53 | 43,1 | 115 | 43,7 | 0,87 |  | 92 | 82,1 | 203 | 86,8 | 0,239 |  | 47 | 42 | 106 | 45,3 | 0,599 |
| Controls | 21 | 17,1 | 37 | 14,1 |  |  | 70 | 56,9 | 148 | 56,3 |  |  | 20 | 17,9 | 31 | 13,2 |  |  | 65 | 58 | 128 | 54,7 |  |
|  |  |  |  |  |  |  |  |  |  |  |  |  |  |  |  |  |  |  |  |  |  |  |  |
| **rs1800629** |  |  |  |  |  |  |  |  |  |  |  |  |  |  |  |  |  |  |  |  |  |  |  |
| Cases | 121 | 98,4 | 248 | 94,3 | 0,081 |  | 33 | 26,8 | 82 | 31,2 | 0,269 |  | 110 | 98,2 | 223 | 95,3 | 0,176 |  | 30 | 26,8 | 73 | 31,2 | 0,318 |
| Controls | 2 | 1,6 | 15 | 5,7 |  |  | 90 | 73,2 | 181 | 68,8 |  |  | 2 | 1,8 | 11 | 4,7 |  |  | 82 | 73,2 | 161 | 68,8 |  |
|  |  |  |  |  |  |  |  |  |  |  |  |  |  |  |  |  |  |  |  |  |  |  |  |
| **rs1801274** |  |  |  |  |  |  |  |  |  |  |  |  |  |  |  |  |  |  |  |  |  |  |  |
| Cases | 105 | 85,4 | 229 | 87,1 | 0,824 |  | 97 | 78,9 | 199 | 75,7 | 0,447 |  | 97 | 86,6 | 204 | 87,2 | 0,963 |  | 86 | 76,8 | 176 | 75,2 | 0,629 |
| Controls | 18 | 14,6 | 34 | 12,9 |  |  | 26 | 21,1 | 64 | 24,3 |  |  | 15 | 13,4 | 30 | 12,8 |  |  | 26 | 23,2 | 58 | 24,8 |  |
|  |  |  |  |  |  |  |  |  |  |  |  |  |  |  |  |  |  |  |  |  |  |  |  |
| **‘cont (1)** |  |  | **All Cases, DAS28 ≤ 2.6** | | | | | | |  |  |  |  |  | **Ig anti-TNF, DAS28 ≤ 2.6** | | | | | |  |  |  |
|  |  | **Dominant Model** | | |  |  |  | **Recessive Model** | | |  |  |  | **Dominant Model** | | |  |  |  | **Recessive Model** | | |  |
| **SNP** | **Yes (n)** | **Yes (%)** | **No (n)** | **No (%)** | **p** |  | **Yes (n)** | **Yes (%)** | **No (n)** | **No (%)** | **p** |  | **Yes (n)** | **Yes (%)** | **No (n)** | **No (%)** | **p** |  | **Yes (n)** | **Yes (%)** | **No (n)** | **No (%)** | **p** |
| **rs396991** |  |  |  |  |  |  |  |  |  |  |  |  |  |  |  |  |  |  |  |  |  |  |  |
| Cases | 85 | 69,1 | 162 | 61,6 | 0,172 |  | 109 | 88,6 | 226 | 85,9 | 0,63 |  | 78 | 69,6 | 142 | 60,7 | 0,132 |  | 99 | 88,4 | 200 | 85,5 | 0,597 |
| Controls | 38 | 30,9 | 101 | 38,4 |  |  | 14 | 11,4 | 37 | 14,1 |  |  | 34 | 30,4 | 92 | 39,3 |  |  | 13 | 11,6 | 34 | 14,5 |  |
|  |  |  |  |  |  |  |  |  |  |  |  |  |  |  |  |  |  |  |  |  |  |  |  |
| **rs3087456** |  |  |  |  |  |  |  |  |  |  |  |  |  |  |  |  |  |  |  |  |  |  |  |
| Cases | 120 | 97,6 | 252 | 95,8 | 0,527 |  | 45 | 36,6 | 118 | 44,9 | 0,113 |  | 109 | 97,3 | 223 | 95,3 | 0,514 |  | 41 | 36,6 | 103 | 44 | 0,167 |
| Controls | 3 | 2,4 | 11 | 4,2 |  |  | 78 | 63,4 | 145 | 55,1 |  |  | 3 | 2,7 | 11 | 4,7 |  |  | 71 | 63,4 | 131 | 56 |  |
|  |  |  |  |  |  |  |  |  |  |  |  |  |  |  |  |  |  |  |  |  |  |  |  |
| **rs1061622** |  |  |  |  |  |  |  |  |  |  |  |  |  |  |  |  |  |  |  |  |  |  |  |
| Cases | 114 | 92,7 | 252 | 95,8 | 0,254 |  | 47 | 38,2 | 108 | 41,1 | 0,358 |  | 103 | 92 | 224 | 95,7 | 0,185 |  | 44 | 39,3 | 100 | 42,7 | 0,377 |
| Controls | 9 | 7,3 | 11 | 4,2 |  |  | 76 | 61,8 | 155 | 58,9 |  |  | 9 | 8 | 10 | 4,3 |  |  | 68 | 60,7 | 134 | 57,3 |  |
|  |  |  |  |  |  |  |  |  |  |  |  |  |  |  |  |  |  |  |  |  |  |  |  |
| **rs2004640** |  |  |  |  |  |  |  |  |  |  |  |  |  |  |  |  |  |  |  |  |  |  |  |
| Cases | 92 | 74,8 | 188 | 71,5 | 0,522 |  | 91 | 74 | 216 | 82,1 | 0,087 |  | 84 | 75 | 170 | 72,6 | 0,588 |  | 83 | 74,1 | 191 | 81,6 | 0,125 |
| Controls | 31 | 25,2 | 75 | 28,5 |  |  | 32 | 26 | 47 | 17,9 |  |  | 28 | 25 | 64 | 27,4 |  |  | 29 | 25,9 | 43 | 18,4 |  |
|  |  |  |  |  |  |  |  |  |  |  |  |  |  |  |  |  |  |  |  |  |  |  |  |
| **rs10954213** |  |  |  |  |  |  |  |  |  |  |  |  |  |  |  |  |  |  |  |  |  |  |  |
| Cases | 98 | 79,7 | 235 | 89,4 | **0,026** |  | 70 | 56,9 | 166 | 63,1 | 0,306 |  | 88 | 78,6 | 209 | 89,3 | **0,015** |  | 65 | 58 | 149 | 63,7 | 0,403 |
| Controls | 25 | 20,3 | 28 | 10,6 |  |  | 53 | 43,1 | 97 | 36,9 |  |  | 24 | 21,4 | 25 | 10,7 |  |  | 47 | 42 | 85 | 36,3 |  |
|  |  |  |  |  |  |  |  |  |  |  |  |  |  |  |  |  |  |  |  |  |  |  |  |
| **rs3890745** |  |  |  |  |  |  |  |  |  |  |  |  |  |  |  |  |  |  |  |  |  |  |  |
| Cases | 105 | 85,4 | 242 | 92 | **0,034** |  | 67 | 54,5 | 136 | 51,7 | 0,768 |  | 94 | 83,9 | 214 | 91,5 | **0,027** |  | 64 | 57,1 | 117 | 50 | 0,309 |
| Controls | 18 | 14,6 | 21 | 8 |  |  | 56 | 45,5 | 127 | 48,3 |  |  | 18 | 16,1 | 20 | 8,5 |  |  | 48 | 42,9 | 117 | 50 |  |
|  |  |  |  |  |  |  |  |  |  |  |  |  |  |  |  |  |  |  |  |  |  |  |  |
| **rs2476601** |  |  |  |  |  |  |  |  |  |  |  |  |  |  |  |  |  |  |  |  |  |  |  |
| Cases | 120 | 97,6 | 257 | 97,7 | 0,789 |  | 46 | 37,4 | 87 | 33,1 | 0,325 |  | 109 | 97,3 | 228 | 97,4 | 0,804 |  | 39 | 34,8 | 72 | 30,8 | 0,374 |
| Controls | 3 | 2,4 | 6 | 2,3 |  |  | 77 | 62,6 | 176 | 66,9 |  |  | 3 | 2,7 | 6 | 2,6 |  |  | 73 | 65,2 | 162 | 69,2 |  |
|  |  |  |  |  |  |  |  |  |  |  |  |  |  |  |  |  |  |  |  |  |  |  |  |
| **rs2816316** |  |  |  |  |  |  |  |  |  |  |  |  |  |  |  |  |  |  |  |  |  |  |  |
| Cases | 119 | 96,7 | 256 | 97,3 | 0,654 |  | 46 | 37,4 | 88 | 33,5 | 0,492 |  | 109 | 97,3 | 227 | 97 | 1 |  | 41 | 36,6 | 77 | 32,9 | 0,519 |
| Controls | 4 | 3,3 | 7 | 2,7 |  |  | 77 | 62,6 | 175 | 66,5 |  |  | 3 | 2,7 | 7 | 3 |  |  | 71 | 63,4 | 157 | 67,1 |  |
|  |  |  |  |  |  |  |  |  |  |  |  |  |  |  |  |  |  |  |  |  |  |  |  |
|  |  |  |  |  |  |  |  |  |  |  |  |  |  |  |  |  |  |  |  |  |  |  |  |
|  |  |  |  |  |  |  |  |  |  |  |  |  |  |  |  |  |  |  |  |  |  |  |  |
| **‘cont (2)** |  |  | **All Cases, DAS28 ≤ 2.6** | | | | | | |  |  |  |  |  | **Ig anti-TNF, DAS28 ≤ 2.6** | | | | | |  |  |  |
|  |  | **Dominant Model** | | |  |  |  | **Recessive Model** | | |  |  |  | **Dominant Model** | | |  |  |  | **Recessive Model** | | |  |
| **SNP** | **Yes (n)** | **Yes (%)** | **No (n)** | **No (%)** | **p** |  | **Yes (n)** | **Yes (%)** | **No (n)** | **No (%)** | **p** |  | **Yes (n)** | **Yes (%)** | **No (n)** | **No (%)** | **p** |  | **Yes (n)** | **Yes (%)** | **No (n)** | **No (%)** | **p** |
| **rs842647** |  |  |  |  |  |  |  |  |  |  |  |  |  |  |  |  |  |  |  |  |  |  |  |
| Cases | 113 | 91,9 | 234 | 89 | 0,509 |  | 67 | 54,5 | 142 | 54 | 0,8 |  | 103 | 92 | 208 | 88,9 | 0,557 |  | 61 | 54,5 | 128 | 54,7 | 0,901 |
| Controls | 10 | 8,1 | 29 | 11 |  |  | 56 | 45,5 | 121 | 46 |  |  | 9 | 8 | 26 | 11,1 |  |  | 51 | 45,5 | 106 | 45,3 |  |
|  |  |  |  |  |  |  |  |  |  |  |  |  |  |  |  |  |  |  |  |  |  |  |  |
| **rs917997** |  |  |  |  |  |  |  |  |  |  |  |  |  |  |  |  |  |  |  |  |  |  |  |
| Cases | 118 | 95,9 | 246 | 93,5 | 0,364 |  | 54 | 43,9 | 121 | 46 | 0,743 |  | 107 | 95,5 | 219 | 93,6 | 0,482 |  | 49 | 43,8 | 109 | 46,6 | 0,736 |
| Controls | 5 | 4,1 | 17 | 6,5 |  |  | 69 | 56,1 | 142 | 54 |  |  | 5 | 4,5 | 15 | 6,4 |  |  | 63 | 56,3 | 125 | 53,4 |  |
|  |  |  |  |  |  |  |  |  |  |  |  |  |  |  |  |  |  |  |  |  |  |  |  |
| **rs7574865** |  |  |  |  |  |  |  |  |  |  |  |  |  |  |  |  |  |  |  |  |  |  |  |
| Cases | 117 | 95,1 | 250 | 95,1 | 0,977 |  | 54 | 43,9 | 101 | 38,4 | 0,279 |  | 106 | 94,6 | 222 | 94,9 | 0,852 |  | 48 | 42,9 | 89 | 38 | 0,365 |
| Controls | 6 | 4,9 | 13 | 4,9 |  |  | 69 | 56,1 | 162 | 61,6 |  |  | 6 | 5,4 | 12 | 5,1 |  |  | 64 | 57,1 | 145 | 62 |  |
|  |  |  |  |  |  |  |  |  |  |  |  |  |  |  |  |  |  |  |  |  |  |  |  |
| **rs6441961** |  |  |  |  |  |  |  |  |  |  |  |  |  |  |  |  |  |  |  |  |  |  |  |
| Cases | 110 | 89,4 | 238 | 90,5 | 0,624 |  | 57 | 46,3 | 146 | 55,5 | 0,188 |  | 101 | 90,2 | 212 | 90,6 | 0,848 |  | 50 | 44,6 | 129 | 55,1 | 0,118 |
| Controls | 13 | 10,6 | 25 | 9,5 |  |  | 66 | 53,7 | 117 | 44,5 |  |  | 11 | 9,8 | 22 | 9,4 |  |  | 62 | 55,4 | 105 | 44,9 |  |
|  |  |  |  |  |  |  |  |  |  |  |  |  |  |  |  |  |  |  |  |  |  |  |  |
| **rs17810546** |  |  |  |  |  |  |  |  |  |  |  |  |  |  |  |  |  |  |  |  |  |  |  |
| Cases | 123 | 100 | 258 | 98,1 | 0,178 |  | 25 | 20,3 | 52 | 19,8 | 0,747 |  | 112 | 100 | 230 | 98,3 | 0,224 |  | 23 | 20,5 | 45 | 19,2 | 0,677 |
| Controls | 0 | 0 | 5 | 1,9 |  |  | 98 | 79,7 | 211 | 80,2 |  |  | 0 | 0 | 4 | 1,7 |  |  | 89 | 79,5 | 189 | 80,8 |  |
|  |  |  |  |  |  |  |  |  |  |  |  |  |  |  |  |  |  |  |  |  |  |  |  |
| **rs9811792** |  |  |  |  |  |  |  |  |  |  |  |  |  |  |  |  |  |  |  |  |  |  |  |
| Cases | 100 | 81,3 | 223 | 84,8 | 0,344 |  | 87 | 70,7 | 173 | 65,8 | 0,417 |  | 90 | 80,4 | 199 | 85 | 0,225 |  | 78 | 69,6 | 155 | 66,2 | 0,563 |
| Controls | 23 | 18,7 | 40 | 15,2 |  |  | 36 | 29,3 | 90 | 34,2 |  |  | 22 | 19,6 | 35 | 15 |  |  | 34 | 30,4 | 79 | 33,8 |  |
|  |  |  |  |  |  |  |  |  |  |  |  |  |  |  |  |  |  |  |  |  |  |  |  |
| **rs1464510** |  |  |  |  |  |  |  |  |  |  |  |  |  |  |  |  |  |  |  |  |  |  |  |
| Cases | 88 | 71,5 | 216 | 82,1 | **0,016** |  | 92 | 74,8 | 180 | 68,4 | 0,232 |  | 80 | 71,4 | 194 | 82,9 | **0,015** |  | 85 | 75,9 | 165 | 70,5 | 0,31 |
| Controls | 35 | 28,5 | 47 | 17,9 |  |  | 31 | 25,2 | 83 | 31,6 |  |  | 32 | 28,6 | 40 | 17,1 |  |  | 27 | 24,1 | 69 | 29,5 |  |
|  |  |  |  |  |  |  |  |  |  |  |  |  |  |  |  |  |  |  |  |  |  |  |  |
| **rs6822844** |  |  |  |  |  |  |  |  |  |  |  |  |  |  |  |  |  |  |  |  |  |  |  |
| Cases | 121 | 98,4 | 256 | 97,3 | 0,619 |  | 28 | 22,8 | 58 | 22,1 | 0,889 |  | 110 | 98,2 | 227 | 97 | 0,609 |  | 25 | 22,3 | 52 | 22,2 | 0,825 |
| Controls | 2 | 1,6 | 7 | 2,7 |  |  | 95 | 77,2 | 205 | 77,9 |  |  | 2 | 1,8 | 7 | 3 |  |  | 87 | 77,7 | 182 | 77,8 |  |
|  |  |  |  |  |  |  |  |  |  |  |  |  |  |  |  |  |  |  |  |  |  |  |  |
|  |  |  |  |  |  |  |  |  |  |  |  |  |  |  |  |  |  |  |  |  |  |  |  |
|  |  |  |  |  |  |  |  |  |  |  |  |  |  |  |  |  |  |  |  |  |  |  |  |
| **‘cont (3)** |  |  | **All Cases, DAS28 ≤ 2.6** | | | | | | |  |  |  |  |  | **Ig anti-TNF, DAS28 ≤ 2.6** | | | | | |  |  |  |
|  |  | **Dominant Model** | | |  |  |  | **Recessive Model** | | |  |  |  | **Dominant Model** | | |  |  |  | **Recessive Model** | | |  |
| **SNP** | **Yes (n)** | **Yes (%)** | **No (n)** | **No (%)** | **p** |  | **Yes (n)** | **Yes (%)** | **No (n)** | **No (%)** | **p** |  | **Yes (n)** | **Yes (%)** | **No (n)** | **No (%)** | **p** |  | **Yes (n)** | **Yes (%)** | **No (n)** | **No (%)** | **p** |
| **rs2327832** |  |  |  |  |  |  |  |  |  |  |  |  |  |  |  |  |  |  |  |  |  |  |  |
| Cases | 119 | 96,7 | 249 | 94,7 | 0,413 |  | 52 | 42,3 | 80 | 30,4 | **0,018** |  | 108 | 96,4 | 222 | 94,9 | 0,522 |  | 46 | 41,1 | 68 | 29,1 | **0,019** |
| Controls | 4 | 3,3 | 14 | 5,3 |  |  | 71 | 57,7 | 183 | 69,6 |  |  | 4 | 3,6 | 12 | 5,1 |  |  | 66 | 58,9 | 166 | 70,9 |  |
|  |  |  |  |  |  |  |  |  |  |  |  |  |  |  |  |  |  |  |  |  |  |  |  |
| **rs10499194** |  |  |  |  |  |  |  |  |  |  |  |  |  |  |  |  |  |  |  |  |  |  |  |
| Cases | 118 | 95,9 | 241 | 91,6 | 0,216 |  | 56 | 45,5 | 121 | 46 | 0,734 |  | 107 | 95,5 | 214 | 91,5 | 0,257 |  | 52 | 46,4 | 109 | 46,6 | 0,798 |
| Controls | 5 | 4,1 | 22 | 8,4 |  |  | 67 | 54,5 | 142 | 54 |  |  | 5 | 4,5 | 20 | 8,5 |  |  | 60 | 53,6 | 125 | 53,4 |  |
|  |  |  |  |  |  |  |  |  |  |  |  |  |  |  |  |  |  |  |  |  |  |  |  |
| **rs1738074** |  |  |  |  |  |  |  |  |  |  |  |  |  |  |  |  |  |  |  |  |  |  |  |
| Cases | 105 | 85,4 | 223 | 84,8 | 0,759 |  | 83 | 67,5 | 168 | 63,9 | 0,483 |  | 100 | 89,3 | 200 | 85,5 | 0,318 |  | 73 | 65,2 | 150 | 64,1 | 0,794 |
| Controls | 18 | 14,6 | 40 | 15,2 |  |  | 40 | 32,5 | 95 | 36,1 |  |  | 12 | 10,7 | 34 | 14,5 |  |  | 39 | 34,8 | 84 | 35,9 |  |
|  |  |  |  |  |  |  |  |  |  |  |  |  |  |  |  |  |  |  |  |  |  |  |  |
| **rs42041** |  |  |  |  |  |  |  |  |  |  |  |  |  |  |  |  |  |  |  |  |  |  |  |
| Cases | 114 | 92,7 | 254 | 96,6 | 0,064 |  | 53 | 43,1 | 111 | 42,2 | 0,601 |  | 104 | 92,9 | 226 | 96,6 | 0,098 |  | 47 | 42 | 93 | 39,7 | 0,519 |
| Controls | 9 | 7,3 | 9 | 3,4 |  |  | 70 | 56,9 | 152 | 57,8 |  |  | 8 | 7,1 | 8 | 3,4 |  |  | 65 | 58 | 141 | 60,3 |  |
|  |  |  |  |  |  |  |  |  |  |  |  |  |  |  |  |  |  |  |  |  |  |  |  |
| **rs2812378** |  |  |  |  |  |  |  |  |  |  |  |  |  |  |  |  |  |  |  |  |  |  |  |
| Cases | 109 | 88,6 | 231 | 87,8 | 0,882 |  | 74 | 60,2 | 174 | 66,2 | 0,231 |  | 98 | 87,5 | 205 | 87,6 | 0,974 |  | 68 | 60,7 | 156 | 66,7 | 0,239 |
| Controls | 14 | 11,4 | 32 | 12,2 |  |  | 49 | 39,8 | 89 | 33,8 |  |  | 14 | 12,5 | 29 | 12,4 |  |  | 44 | 39,3 | 78 | 33,3 |  |
|  |  |  |  |  |  |  |  |  |  |  |  |  |  |  |  |  |  |  |  |  |  |  |  |
| **rs3761847** |  |  |  |  |  |  |  |  |  |  |  |  |  |  |  |  |  |  |  |  |  |  |  |
| Cases | 102 | 82,9 | 220 | 83,7 | 0,984 |  | 95 | 77,2 | 185 | 70,3 | 0,127 |  | 91 | 81,3 | 200 | 85,5 | 0,465 |  | 87 | 77,7 | 162 | 69,2 | 0,083 |
| Controls | 21 | 17,1 | 43 | 16,3 |  |  | 28 | 22,8 | 78 | 29,7 |  |  | 21 | 18,8 | 34 | 14,5 |  |  | 25 | 22,3 | 72 | 30,8 |  |
|  |  |  |  |  |  |  |  |  |  |  |  |  |  |  |  |  |  |  |  |  |  |  |  |
| **rs4750316** |  |  |  |  |  |  |  |  |  |  |  |  |  |  |  |  |  |  |  |  |  |  |  |
| Cases | 120 | 97,6 | 254 | 96,6 | 0,557 |  | 43 | 35 | 102 | 38,8 | 0,515 |  | 109 | 97,3 | 226 | 96,6 | 0,679 |  | 39 | 34,8 | 86 | 36,8 | 0,728 |
| Controls | 3 | 2,4 | 9 | 3,4 |  |  | 80 | 65 | 161 | 61,2 |  |  | 3 | 2,7 | 8 | 3,4 |  |  | 73 | 65,2 | 148 | 63,2 |  |
|  |  |  |  |  |  |  |  |  |  |  |  |  |  |  |  |  |  |  |  |  |  |  |  |
| **rs1678542** |  |  |  |  |  |  |  |  |  |  |  |  |  |  |  |  |  |  |  |  |  |  |  |
| Cases | 108 | 87,8 | 241 | 91,6 | 0,236 |  | 74 | 60,2 | 148 | 56,3 | 0,524 |  | 98 | 87,5 | 214 | 91,5 | 0,261 |  | 66 | 58,9 | 132 | 56,4 | 0,788 |
| Controls | 15 | 12,2 | 22 | 8,4 |  |  | 49 | 39,8 | 115 | 43,7 |  |  | 14 | 12,5 | 20 | 8,5 |  |  | 46 | 41,1 | 102 | 43,6 |  |
|  |  |  |  |  |  |  |  |  |  |  |  |  |  |  |  |  |  |  |  |  |  |  |  |
|  |  |  |  |  |  |  |  |  |  |  |  |  |  |  |  |  |  |  |  |  |  |  |  |
|  |  |  |  |  |  |  |  |  |  |  |  |  |  |  |  |  |  |  |  |  |  |  |  |
| **‘cont (4)** |  |  | **All Cases, DAS28 ≤ 2.6** | | | | | | |  |  |  |  |  | **Ig anti-TNF, DAS28 ≤ 2.6** | | | | | |  |  |  |
|  |  | **Dominant Model** | | |  |  |  | **Recessive Model** | | |  |  |  | **Dominant Model** | | |  |  |  | **Recessive Model** | | |  |
| **SNP** | **Yes (n)** | **Yes (%)** | **No (n)** | **No (%)** | **p** |  | **Yes (n)** | **Yes (%)** | **No (n)** | **No (%)** | **p** |  | **Yes (n)** | **Yes (%)** | **No (n)** | **No (%)** | **p** |  | **Yes (n)** | **Yes (%)** | **No (n)** | **No (%)** | **p** |
| **rs3184504** |  |  |  |  |  |  |  |  |  |  |  |  |  |  |  |  |  |  |  |  |  |  |  |
| Cases | 94 | 76,4 | 200 | 76 | 0,549 |  | 89 | 72,4 | 185 | 70,3 | 0,825 |  | 86 | 76,8 | 175 | 74,8 | 0,44 |  | 79 | 70,5 | 167 | 71,4 | 0,802 |
| Controls | 29 | 23,6 | 63 | 24 |  |  | 34 | 27,6 | 78 | 29,7 |  |  | 26 | 23,2 | 59 | 25,2 |  |  | 33 | 29,5 | 67 | 28,6 |  |
|  |  |  |  |  |  |  |  |  |  |  |  |  |  |  |  |  |  |  |  |  |  |  |  |
| **rs4810485** |  |  |  |  |  |  |  |  |  |  |  |  |  |  |  |  |  |  |  |  |  |  |  |
| Cases | 116 | 94,3 | 256 | 97,3 | 0,154 |  | 56 | 45,5 | 104 | 39,5 | 0,319 |  | 106 | 94,6 | 228 | 97,4 | 0,185 |  | 53 | 47,3 | 89 | 38 | 0,116 |
| Controls | 7 | 5,7 | 7 | 2,7 |  |  | 67 | 54,5 | 159 | 60,5 |  |  | 6 | 5,4 | 6 | 2,6 |  |  | 59 | 52,7 | 145 | 62 |  |
|  |  |  |  |  |  |  |  |  |  |  |  |  |  |  |  |  |  |  |  |  |  |  |  |
| **rs3218253** |  |  |  |  |  |  |  |  |  |  |  |  |  |  |  |  |  |  |  |  |  |  |  |
| Cases | 113 | 91,9 | 238 | 90,5 | 0,508 |  | 72 | 58,5 | 124 | 47,1 | **0,042** |  | 103 | 92 | 212 | 90,6 | 0,539 |  | 66 | 58,9 | 114 | 48,7 | 0,081 |
| Controls | 10 | 8,1 | 25 | 9,5 |  |  | 51 | 41,5 | 139 | 52,9 |  |  | 9 | 8 | 22 | 9,4 |  |  | 46 | 41,1 | 120 | 51,3 |  |
|  |  |  |  |  |  |  |  |  |  |  |  |  |  |  |  |  |  |  |  |  |  |  |  |
| **rs12252317** |  |  |  |  |  |  |  |  |  |  |  |  |  |  |  |  |  |  |  |  |  |  |  |
| Cases | 122 | 99,2 | 259 | 98,5 | 0,647 |  | 14 | 11,4 | 46 | 17,5 | 0,166 |  | 111 | 99,1 | 231 | 98,7 | 0,7 |  | 12 | 10,7 | 39 | 16,7 | 0,157 |
| Controls | 1 | 0,8 | 4 | 1,5 |  |  | 109 | 88,6 | 217 | 82,5 |  |  | 1 | 0,9 | 3 | 1,3 |  |  | 100 | 89,3 | 195 | 83,3 |  |
|  |  |  |  |  |  |  |  |  |  |  |  |  |  |  |  |  |  |  |  |  |  |  |  |
| **rs9770242** |  |  |  |  |  |  |  |  |  |  |  |  |  |  |  |  |  |  |  |  |  |  |  |
| Cases | 114 | 92,7 | 246 | 93,5 | 0,845 |  | 56 | 45,5 | 115 | 43,7 | 0,717 |  | 103 | 92 | 218 | 93,2 | 0,741 |  | 51 | 45,5 | 110 | 47 | 0,934 |
| Controls | 9 | 7,3 | 17 | 6,5 |  |  | 67 | 54,5 | 148 | 56,3 |  |  | 9 | 8 | 16 | 6,8 |  |  | 61 | 54,5 | 124 | 53 |  |
|  |  |  |  |  |  |  |  |  |  |  |  |  |  |  |  |  |  |  |  |  |  |  |  |
| **PBEF1543** |  |  |  |  |  |  |  |  |  |  |  |  |  |  |  |  |  |  |  |  |  |  |  |
| Cases | 112 | 91,1 | 245 | 93,2 | 0,52 |  | 55 | 44,7 | 118 | 44,9 | 0,707 |  | 101 | 90,2 | 220 | 94 | 0,251 |  | 48 | 42,9 | 107 | 45,7 | 0,436 |
| Controls | 11 | 8,9 | 18 | 6,8 |  |  | 68 | 55,3 | 145 | 55,1 |  |  | 11 | 9,8 | 14 | 6 |  |  | 64 | 57,1 | 127 | 54,3 |  |
|  |  |  |  |  |  |  |  |  |  |  |  |  |  |  |  |  |  |  |  |  |  |  |  |
| **rs16986050** |  |  |  |  |  |  |  |  |  |  |  |  |  |  |  |  |  |  |  |  |  |  |  |
| Cases | 121 | 98,4 | 254 | 96,6 | 0,297 |  | 29 | 23,6 | 74 | 28,1 | 0,344 |  | 110 | 98,2 | 228 | 97,4 | 0,554 |  | 27 | 24,1 | 67 | 28,6 | 0,42 |
| Controls | 2 | 1,6 | 9 | 3,4 |  |  | 94 | 76,4 | 189 | 71,9 |  |  | 2 | 1,8 | 6 | 2,6 |  |  | 85 | 75,9 | 167 | 71,4 |  |

**Legend to Tables**

**Table S2 –** Number and proportion of events (e.g. clinical remission) in patients carrying the selected genotypes (cases) or not (controls). Under the dominant model, cases were constituted by patients carrying at least 1 copy of the mutant allele. Under the recessive model, cases were constituted by patients carrying both copies of the mutant allele. P values are calculated via the log-rank test statistic with 1 degree of freedom. The analysis was performed either considering the patients who received treatment with anti-tumor necrosis factor antibodies (Ig anti-TNF) (e.g. infliximab and/or adalimumab) or considering all the patients, either treated with Ig anti-TNF or anti-TNF receptors (e.g. etanercept).
